# An evaluation of Astragali Radix with different growth patterns and years, based on a new multidimensional comparison method

**DOI:** 10.3389/fpls.2024.1368135

**Published:** 2024-02-29

**Authors:** Yapeng Wang, Changsheng Yuan, Jiachen Zhao, Yunxiang Liu, Chunfang Tian, Jinxiu Qian, Tiegui Nan, Liping Kang, Yanmeng Liu, Zhilai Zhan, Luqi Huang

**Affiliations:** State Key Laboratory for Quality Ensurance and Sustainable Use of Dao-di Herbs, National Resource Center for Chinese Materia Medica, China Academy of Chinese Medica Sciences, Beijing, China

**Keywords:** Astragali Radix, growth patterns, growth years, quality difference, imitated-wild cultivation

## Abstract

**Introduction:**

With the depletion of wild Astragali Radix (WA) resources, imitated-wild Astragali Radix (IWA) and cultivated Astragali Radix (CA) have become the main products of Astragali Radix. However, the quality differences of three growth patterns (WA, IWA, CA) and different growth years of Astragali Radix have not been fully characterized, leading to a lack of necessary scientific evidence for their use as substitutes for WA.

**Methods:**

We innovatively proposed a multidimensional evaluation method that encompassed traits, microstructure, cell wall components, saccharides, and pharmacodynamic compounds, to comprehensively explain the quality variances among different growth patterns and years of Astragali Radix.

**Results and discussion:**

Our study showed that the quality of IWA and WA was comparatively similar, including evaluation indicators such as apparent color, sectional structure and odor, thickness of phellem, diameter and number of vessels, morphology of phloem and xylem, and the levels and ratios of cellulose, hemicellulose, lignin, sucrose, starch, water-soluble polysaccharides, total-saponins. However, the content of sucrose, starch and sorbose in CA was significantly higher than WA, and the diameter and number of vessels, total-flavonoids content were lower than WA, indicating significant quality differences between CA and WA. Hence, we suggest that IWA should be used as a substitute for WA instead of CA. As for the planting years of IWA, our results indicated that IWA aged 1-32 years could be divided into three stages according to their quality change: rapid growth period (1-5 years), stable growth period (6-20 years), and elderly growth period (25-32 years). Among these, 6-20 years old IWA exhibited consistent multidimensional comparative results, showcasing elevated levels of key active components such as water-soluble polysaccharides, flavonoids, and saponins. Considering both the quality and cultivation expenses of IWA, we recommend a cultivation duration of 6-8 years for growers. In conclusion, we established a novel multidimensional evaluation method to systematically characterize the quality of Astragali Radix, and provided a new scientific perspective for the artificial cultivation and quality assurance of Astragali Radix.

## 1 Introduction

Astragali Radix (AR) is derived from the roots of *Astragalus membranaceus* (Fisch.) Bge. var. *mongholicus* (Bge.) Hsiao or *A. membranaceus* (Fisch.) Bge. (China, 2020). It was one of the most commonly used traditional medicines in clinic and a frequent food additive. AR was reported to have a wide range of pharmacological effects, such as immune modulation, anti-oxidation, anti-inflammation, anti-tumorigenesis and anti-myocardial ischemia. Its main active components were water-soluble polysaccharides, isoflavones and saponins (Abd Elkader et al., 2022). In ancient China, AR used was wild Astragalus Radix (WA) (Zhao et al., 2022), but nowadays the resources of WA have been seriously insufficient. Imitated-wild Astragali Radix (IWA) and cultivated Astragali Radix (CA) have become the major products in the market (Qin et al., 2016). IWA was planted in the wild mountain slopes and harvested in 6 years, while CA was cultivated in conventional farmland and harvested in 2 years with the implementation of artificial management measures (Zhang et al., 2022).

The quality difference between WA, IWA, and CA is the most urgent problem to be solved in the production and use of AR. The majority of research has focused on secondary metabolites. For example, fourteen compounds (calycosin-7-*O*-*β*-D-glucoside, ononin, formononetin, calycosin, astragaloside I-IV, etc.) were quantified in order to distinguish IWA and CA (Wang et al., 2020). Forty-two flavonoids and eight saponins were found to differentiate IWA and CA by metabolomics (Yang et al., 2022a). However, evaluating the quality of AR through secondary metabolites alone is insufficient. Traits, microstructure, cell wall components and primary metabolites are all indispensable aspects in the quality evaluation of AR. The strength of the environmental stress is a crucial factor determining the quality of medicinal plants (Huang and Guo, 2007; Ma et al., 2022). Changes in traits and microstructure are the macroscopic and microscopic manifestations of a plant’s response to stress, directly expressing the quality of AR. Variations in the contents of cell wall components such as cellulose, hemicellulose and lignin could alter the characteristics of plant cell walls to cope with stressors such as drought, low temperatures, salinity, pests, and diseases (Yang et al., 2023b). Simultaneously, changes in cell wall components also served as a significant material basis for explaining the microstructure and external characteristics changes of AR (Zhao et al., 2024). Primary metabolites such as free monosaccharides, sucrose, starch, and water-soluble polysaccharides, as fundamental substances for the growth and development of AR, exhibited direct correlations with the stress response and quality changes of Astragalus (Wang et al., 2022a). Regarding the relationship between various growth modes and AR quality changes, it is unreliable to assess the quality of WA, IWA, and CA solely based on secondary metabolites. Thus, we suggest developing a multidimensional assessment method that includes aspects of traits, microstructure, cell wall components, content of primary and secondary metabolites. The objective is to systematically scrutinize three growth patterns of AR, effectively addressing the current research voids.

Moreover, IWA was a simulated cultivation of WA, but WA usually grew for more than 10 years, whereas IWA was mainly 6 years old. Investigating the quality changes in IWA with the cultivation period has a great significance for determining the optimal planting duration and ensuring high-quality production of IWA. However, to date, related studies were concentrated on low-year-old (< 10 years). There is a lack of studies on high-year-old (> 10 years) IWA. For example, in IWA aged 2-5 years, there was an increase in diameter, root length, and seed quality (Wang et al., 2021). The highest content of saponins and flavonoids was found in the IWA aged 3-4 years (Gao et al., 2018). To ensure the scientific rationality of IWA cultivation, it is imperative to comprehensively characterize the patterns of quality changes in high-year-old IWA, considering factors such as traits, microstructure, cell wall fractions, primary and secondary metabolite contents. This effort aims to address the existing research gaps on high-year-old IWA and offer scientific guidance for their cultivation.

Therefore, we plan to establish a novel multidimensional evaluation approach to systematically compare three different growth modes AR (WA, IWA, CA), in order to provide an important scientific guideline for the use of IWA and CA as a substitute for the WA. The multidimensional evaluation method mainly includes traits, microstructure, cell wall components, saccharides, and pharmacodynamic compounds. Furthermore, since there is no research on the quality of high-year-old (> 10 years) IWA, we intend to use the multidimensional method to analyze the quality changes of 1-32 years IWA. This is helpful and vital to give the scientific planting timeframe range of IWA.

## 2 Materials and methods

### 2.1 Plant materials, chemicals and reagents

A total of 19 batches of AR samples were collected in this study, including different growth patterns of AR (WA, IWA, CA) and different growth years of IWA (1-9, 11, 15, 18, 19, 20, 25, 26, 32 years). All of them were identified as the roots of *A. membranaceus* var. *mongholicus* by Professor Zhilai Zhan. The growth year of all samples was provided by the farmers where the samples were collected, and was verified by observing the layers of growth ring in AR. Each sample was dried at 60 °C for 48 h and then grounded to powder by Mixer Mill MM500 Vario (Retsch, Germany). All sample powders were pulverized to pass through a 65-mesh sieve. Detailed information of the samples was presented in **Supplementary Table 1**.

Sorbose, glucose, sucrose, calycosin-7-*O-β*-D-glucoside, ononin, methylnissolin-3-*O*-glucoside, calycosin, formononetin, isomucronulatol, astragaloside IV, astragaloside III, astragaloside II, and astragaloside I (> 98%) were purchased from Beijing Beite Renkang Biopharmaceutical Technology Co., Ltd. Their structures were shown in **Supplementary Figure 1**. Pure water was distilled water provided by Watson’s Company, and all other reagents were analytical grade.

### 2.2 Traits and microstructure observation

An electronic camera (Canon EOS-800D, Lens EF 24-105 mm f/4 L IS USM, Macro Lens EF 100mm f/2.8 L IS USM, Japan) was used to take images of AR as a whole, local features, folded surfaces and cross sections. Measured the length of AR and the diameter at 3 cm below the residual part of stem. Compared and described the differences in traits among the groups.

Fresh Astragalus root segments about 7 mm thick were dehydrated with a series of ethanol solutions and fixed in paraffin waxes, processed into permanent sections (10 -15 μm) at -22 °C using a cryo-sectionalizer (Leica CM1860, Germany) and stained with safranin and fast green. The microstructural differences between the groups of AR were observed and analyzed under a light microscope (Olympus BX51, Japan).

### 2.3 Quantitative analysis of cell wall components and saccharides

#### 2.3.1 Cellulose, hemicellulose and lignin contents

Using a cellulose analyzer (ANKOM 220, US) and a series of step-by-step treatments including neutral detergent washed, acidic detergent washed, 72% concentrated sulfuric acid washed and residue washed, etc. The cellulose, hemicellulose and lignin contents of the samples were determined using Van Soest’s washed fiber analysis method as a basis (Bender et al., 2016).

#### 2.3.2 Water-soluble polysaccharide and starch contents

Water-soluble polysaccharide and starch content were determined using the anthrone-sulfuric acid method. About 0.1 g of AR powder was weighed accurately in a flask containing 20 ml of 80% ethanol and sonicated at 40 kHz for 60 min. After centrifugation, the insoluble material was retained, and the operation was repeated twice. The residue was added to 20 ml of boiling water and sonicated at 40 kHz for 60 min at 70°C. After centrifugation, 20 ml of boiling water was added and sonicated again, and the filtrate was combined and concentrated to 50 ml to obtain the water-soluble polysaccharide solution to be tested. Then, the filtrate was added to 2 ml of water and placed in boiling water to make starch gelatinization. 2 ml of perchloric acid was added, 5 ml of water was added and centrifuged after sufficient reaction, and the filtrate was combined and the supernatant was fixed to 50 ml after one repetition to obtain the starch solution to be tested. Took 0.2 ml of two solutions, added 0.3 ml of 2% anthrone-ethyl acetate reagent and 3 ml of concentrated sulfuric acid, measured the absorbance at 620 nm for 20 min in a boiling water bath, and then calculated the content with glucose as a reference.

#### 2.3.3 Sucrose, free monosaccharide and extraction contents

0.1 g of AR powder was accurately weighed, 2 ml of 80% methanol was added and sonicated at 40 kHz for 45 min. After centrifuged at 12000 rpm for 10 min, filtered through a 0.2 μm filter membrane to obtain the free monosaccharide sample solution, which was then diluted 10-fold to obtain the sucrose sample solution. The samples were separated using ultra-performance liquid chromatography coupled with a evaporative light-scattering detector (UPLC-ELSD) (Waters Corp., Milford, MA, USA) and a Waters BEH Amide C18 column (2.1 mm×100 mm, 1.8 μm). The mobile phase consisted of 0.1% ammonia-water (solvent A) and 0.1% ammonia-acetonitrile (solvent B), and the gradient was as follows: 18% A for 8 min for free monosaccharides detection; 30% A for 5.5 min for sucrose detection. The flow rate was 0.2 ml/min, and the column temperature was 40°C. The injection volume was 1 μl. The temperature of the drift tube was 50°C, the gain was 500, the gas pressure was 40 psi and the sprayer was in cooling mode. The extraction content was determined according to the method specified in the *Pharmacopoeia of the People’s Republic of China* (China, 2020), using water and 80% ethanol as the extraction solvents.

### 2.4 Quantitative analysis of secondary metabolites

#### 2.4.1 Total-flavonoids and total-saponins contents

Total-flavonoids samples were prepared in the same way as free monosaccharides. The absorbance of the solution was measured at 278 nm, and the total-flavonoids content was calculated with the reference of calycosin-7-*O*-*β*-D-glucoside. Total-saponins: 0.1 g of AR powder was accurately weighed and mixed with 2 ml of methanol, sonicated at 40 kHz for 45 min. 100 μl of supernatant was blown dry under nitrogen, then added with 5% vanillin-ethyl acetate solution of 0.2 ml, perchloric acid of 0.8 ml and water bath at 60°C for 20 minutes. Mixed it with glacial acetic acid of 5 ml and shake well. The absorbance was measured at 560 nm, and the total saponin content was calculated using astragaloside IV as a reference.

#### 2.4.2 Contents of ten main active compounds

The sample powder of AR was weighed accurately at 1.0 g, cold-soaked for 16 h with 40 ml methanol, and ultrasonicated (40 kHz) at 50°C for 1 h. After centrifugation, the solvent was recovered and the residue was accurately dissolved with 2 ml methanol, then the solution of the sample was obtained by filtration of the 0.22 μm membrane. Compounds of AR were separated using UPLC-ELSD and a Waters BEH Amide C18 column (2.1 mm×100 mm, 1.8 μm). The mobile phase consisted of 0.1% formic acid-water (solvent A) and 0.1% formic acid-acetonitrile (solvent B), and the gradient was as follows: 0-3 min, 95-80% A; 3-6 min, 80-74% A; 6-9 min, 74% A; 9-10 min, 74-67% A; 10-11.5 min, 67% B; 11.5-12 min, 67-58% A; 12-16 min, 58% A; 16-16.5 min, 58-35% A; 16.5-28 min, 35-5% A. The flow rate was 0.3 ml/min, the other instrument settings equate to those in 2.3.3. Standard compounds as reference can be found in 2.1.

### 2.5 Statistical analysis

One-way analysis of the variance (ANOVA) or non-parametric test analyses of the content data of AR were analyzed by IBM SPSS Statistics 27.0 (SPSS, Inc., US). Heatmap and hierarchical cluster analysis (HCA) was created using Tbtools-II 2.027 software (Chen et al., 2023a). Principal component analysis (PCA) and partial least-squares discriminant analysis (PLS-DA) were conducted using SIMCA-P 14.0 software (Umerics, Sweden).

## 3 Results and discussions

### 3.1 Multidimensional comparison of WA, IWA and CA

#### 3.1.1 Differences in macroscopic traits

Variances in traits were evident among WA, IWA and CA. Firstly, the diameters of WA and IWA (15-25 mm) were larger than CA (10-15 mm) (**Supplementary Figure 2A**). Regarding root length, the order was IWA > WA > CA (**Supplementary Figure 2B**). IWA tended to grow for a shorter duration compared to WA, yet its root length surpassed that of WA variant. This discrepancy may be attributed to the looser soil conditions in which IWA grow, resulting in less resistance to root elongation.

**Figure 1:**
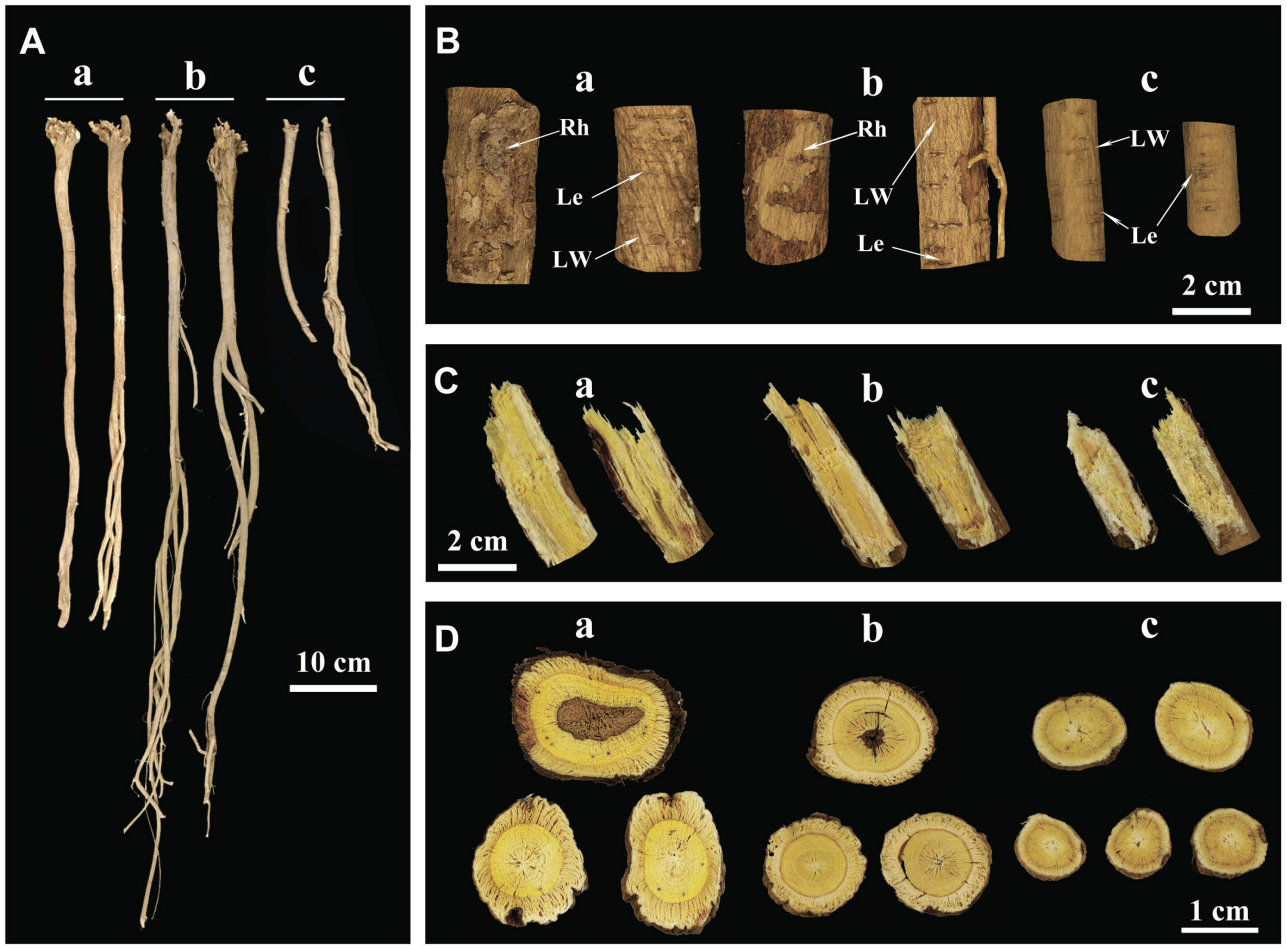
Macroscopic Characteristics of WA, IWA and CA. **(A)** Overall characteristics, **(B)** External features, **(C)** Fracture surfaces, **(D)** Cross-sections. a, WA; b, IWA; c, CA. Rh, Rhytidome; LW, Longitudinal Wrinkles; Le, Lenticel.

Secondly, in terms of external appearance and texture, WA exhibited a straight and elongated physique, with a bright brownish-yellow color and distinct rhytidome (**Figures 1A**-a, **B**-a). IWA had a slightly darker external color, appearing earthy yellow, with rhytidome morphology closely resembling that of WA (**Figures 1A**-b, **B**-b). On the other hand, CA had a light yellow exterior with no rhytidome (**Figures 1A**-c, **B**-c). The color of AR epidermis and the morphology of the rhytidome directly reflected its growth years, as the aging of the periderm tissues leaded to a darker external color and the formation of a rhytidome (Han et al., 2021). Therefore, WA had the most extensive rhytidome, followed by IWA, while CA has not yet formed rhytidome with its shorter growth period. The lenticel served as channels for gas exchange between plant roots and soil, and their quantity was positively correlated with the density of the soil in which the plant was located (Lincoln and Eduardo, 2015). Both WA and CA exhibited numerous and densely distributed lenticel (**Figures 1B**-a, **B**-c), while IWA had fewer lenticel that were more sparsely distributed (**Figure 1B**-b). This suggests that the soil density is greater and the aeration is weaker for WA and CA compared to IWA. In addition to the above characteristics, both WA and IWA displayed noticeable longitudinal wrinkles, while CA lacked. The formation of longitudinal wrinkles in AR was related to the inward concavity of the epidermis caused by parenchymal cavities during dehydration. Therefore, the quantity of longitudinal wrinkles and the degree of concavity could reflect the degree of porosity in the roots of AR. The distinct longitudinal wrinkles in WA and IWA indicated a higher presence of voids in their interiors, contributing to the lightweight texture, whereas CA.

Thirdly, from the perspective of fracture surface characteristics and odor, WA exhibited strong fibrous texture on the fracture surface, with a pronounced beany aroma, a bright yellow color in the xylem, and a white bark layer with a small amount of powder (**Figure 1C**-a). IWA had a fracture surface with a deeper yellow color in the xylem compared to WA, while other features remained largely similar to WA (**Figure 1C**-b). In comparison, the fracture surface of CA showed a pale yellow, fibrous and fragmented xylem, with a higher amount of powder in the phloem and a less pronounced beany aroma (**Figure 1C**-c). The cross-sectional characteristics reflected the macroscopic tissue structure of AR. The fibrous texture indicated the strength or weakness of the plant’s support capacity during growth. The powder on the fracture surface represented starch present in AR. The beany aroma was one of the traditional evaluation indicators, a stronger aroma considered preferable and its chemical basis being the presence of n-hexanal in AR (Sun et al., 2010). Thus it could be inferred that WA and IWA required stronger support for root growth, and had lower starch storage and higher levels of n-hexanal. In contrast, CA experienced less resistance to root growth, had weaker fibrous texture, but exhibited more abundant starch content in the plant.

Finally, the cross-section of WA appeared irregularly round, with a bright yellow xylem displaying growth-ring-like patterns, and a yellow-white phloem with numerous fissures. Some sections of WA xylem exhibited heartwood, and the phloem contained insect holes (**Figure 1D**-a). The cross-section of IWA was approximately round, also featuring growth-ring-like patterns, but with a darker color in xylem compared to WA, and a lighter color in phloem. Other characteristics closely resemble those of WA (**Figure 1D**-b). The cross-section of CA showed a predominantly yellow-brown or light yellow color in the xylem, with the center and phloem both appeared white. Its diameter was slightly smaller than that of WA and IWA (**Figure 1D**-c). The color contrast between the yellow xylem and white phloem in AR constitutes the evaluation criterion ‘JinjingYulan’. Generally, a higher contrast was indicative of a richer content of flavonoids and saponins in AR (Yang et al., 2022b). The presence of heartwood in AR was a natural occurrence in the wood and interxylary cork of the plant (Han et al., 2021), reflecting the higher growth years of WA and IWA.

In summary, WA and IWA exhibited similarities in epidermal layer, longitudinal wrinkles, pore morphology, fracture surface features, aroma, and cross-sectional attributes. However, CA displayed certain distinctions from both. Additionally, the WA samples collected in this study aligned with the characteristics of high-quality AR described in the ancient text “*BenCaoMengQuan*” (Chen, 1988). This suggests that WA samples in this study could represent the AR of historically utilized.

#### 3.1.2 Differences in microstructure

**Figure 2:**
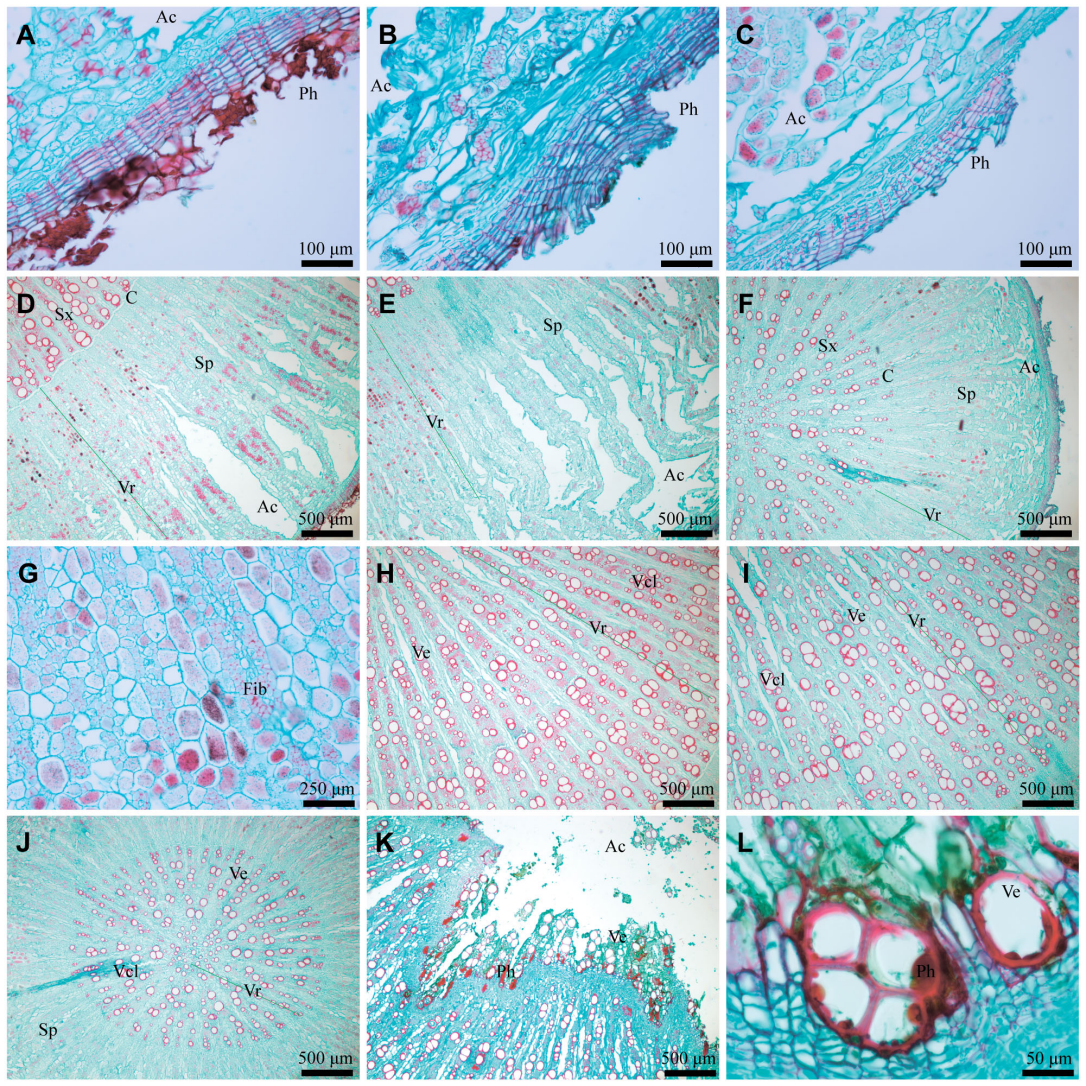
Microstructural characteristics of WA, IWA and CA. **(A–C)** Phellem of WA, IWA, CA; **(D–F)** Secondary-phloem of WA, IWA, CA; **(G)** Fibrocyte of WA, **(H–J)** Secondary-xylem of WA, IWA, CA, **(K)** Heartwood of AR, **(L)** Vessels of heartwood. Ph, Phellem; Ac, Aerenchyma; Sx, Secondary-xylem; C, Cambium; Sp, Secondary-phloem; Vr, Vascular-ray; Fib, Fibrocyte; Ve, Vessel; Vcl, Vessel-cluster.

Microstructure serves as the direct material basis for the characteristics of AR, allowing for a quantitative comparison of differences in its traits. Firstly, the number of phellem cells in WA and IWA ranged mostly from 10 to 20 layers, with a few areas exceeding 30 layers, and there existed a dense red phellem (**Figures 2A, B**). Conversely, the phellem cells in CA were predominantly between 5 and 15 layers (**Figure 2C**). Phellem forms the basis of the periderm and rhytidome of AR. The differences in phellem provide a direct explanation for the abundance of rhytidome in WA and IWA, and the absence of rhytidome in CA.

Secondly, the phloem of WA and IWA exhibited more fractures, with the inner part appearing fragmented, while CA generally lacked fractures. Influenced by it, the bending degree of the vascular rays in WA and IWA were substantial, making it less observable (**Figures 2D, E**), whereas the vascular rays in CA were more distinct (**Figure 2F**). In the phloem of WA and IWA, there were still numerous phellem cell clusters, appeared purple-red in safranin-fast green staining (**Figure 2G**). In comparison, there were fewer phellem cells in CA, with the majority being parenchyma cells or fibrocytes (**Figure 2F**). The phloem served as the primary storage site for saccharides in AR (Chen et al., 2023b), and the quantity of parenchyma cells indicated that CA could store more photosynthetic products than WA and IWA. This characteristic is consistent with the higher content of saccharides in CA, as we observed in 3.1.4.

Finally, the xylems of WA and IWA exhibited clustered distributions of 2-5 vessels, with numbers of xylem ray vessels ranging between 50 and 70 (**Figures 2H, I**). In contrast, the numbers of xylem ray vessels in WA ranged from 20 to 35, with rare vessel groups (**Figure 2J**). Vessels serve as channels for water transport in AR. It could be inferred that WA and IWA exhibit higher water transport capabilities than CA. Additionally, substantial phellem (**Figure 2K**) were observed in the heartwood of WA and IWA, with some vessels showing unidentified fillings (**Figure 2L**). This phenomenon is the microcosmic cause of the programmed xylem death and heartwood of AR (Han et al., 2021). Considering the overall microstructural characteristics of AR, there is a higher resemblance in the phellem, phloem, vessels and xylem between WA and IWA, while CA exhibits discernible differences from both.

#### 3.1.3 Differences in the contents of cell wall components

**Figure 3:**
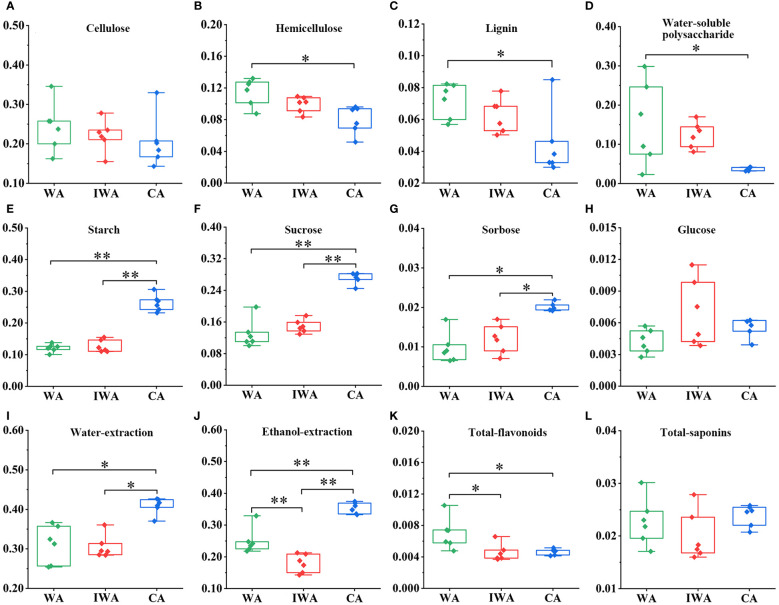
The contents of various chemical compositions in WA, IWA and CA (g/g). **(A)** Cellulose, **(B)** Hemicellulose, **(C)** Lignin, **(D)** Water-soluble polysaccharide, **(E)** Starch, **(F)** Sucrose, **(G)** Sorbose, **(H)** Glucose, **(I)** Water-extraction, **(J)** Ethanol-extraction, **(K)** Total-flavonoids, **(L)** Total-saponins. WA, Wild Astragali Radix; IWA, Imitated-wild Astragali Radix; CA, Cultivated Astragali Radix. **, *p*<0.01; *, *p*<0.05.

Cellulose, hemicellulose, and lignin are main constituents of plant cell walls. They play crucial roles in the normal growth processes of plant roots, including elongation, thickening, branching, and resistance to various environmental stresses. There was no significant difference in cellulose content among WA, IWA, and CA (**Figure 3A**). However, hemicellulose and lignin contents in WA were significantly higher than in CA (**Figures 3B, C**), with IWA falling between both and closer to WA. The elevated hemicellulose content in WA could contribute to higher root hardness and mechanical strength compared to CA (Meents et al., 2018). The increased lignin content of WA could enhance its wood stiffness, water transport capacity, and resistance to diseases, also explaining its greater root strength and straighter plant morphology (Zhao et al., 2021; Wang et al., 2022b). The relatively higher levels of hemicellulose and lignin in WA and IWA were also chemical factors of some differences in traits and microscopic structures, such as the strong fibrous of the fracture surface, the greater number of cross-sectional vessels and lignified cells than CA. In terms of cell wall compositions, IWA exhibited better similarity to WA, indicating stronger stress resistance, while CA showed relatively lower performance.

#### 3.1.4 Differences in the contents of saccharides

The primary products of plant photosynthesis encompass cellulose, hemicellulose, water-soluble polysaccharides, starch, sucrose, monosaccharides and so on. They constituted a significant proportion of the chemical composition in the roots of AR. Firstly, similar to hemicellulose in 3.1.3, the content of water-soluble polysaccharides was notably higher in WA compared to CA, with IWA positioned between them (**Figure 3D**). Water-soluble polysaccharides in AR were reported to exhibit immunomodulatory, anti-insulin resistance, and anti-myocardial ischemia effects (Du et al., 2022). The comparatively higher content of water-soluble polysaccharides in WA and IWA may contribute to a more potent pharmacological effect. Starch, a vital storage substance for the energy produced in AR photosynthesis, reflected the strength of AR’s photosynthetic capacity (Martinez-Vilalta et al., 2016). Starch content in WA and IWA remained at lower levels, whereas CA exhibited a starch content exceeding the former by more than 10%, displaying a highly significant difference (**Figure 3E**). This indicated a robust photosynthetic capacity and vigorous growth and development in CA, while the accumulation of photosynthetic products in WA and IWA was comparatively slower.

Secondly, sucrose was a key primary metabolite involved in signal transduction, resistance to environmental stress, and synthesis of substances such as cellulose, hemicellulose, starch, pectin, and proteins (Yoon et al., 2021). It could directly reflect the strength of AR photosynthetic capabilities, growth and developmental status, and the degree of external environmental stress. The trend in sucrose content closely mirrored that of starch, with sucrose levels in CA exceeding those in WA and IWA by more than 10% (**Figure 3F**). As a direct product of photosynthesis, sucrose was served as the biological precursor for the synthesis of polysaccharides such as cellulose, hemicellulose, water-soluble polysaccharides, and starch (Meents et al., 2018; Li et al., 2021). WA and IWA exhibited lower sucrose and starch content, with higher levels of hemicellulose and water-soluble polysaccharides, indicating their tendency to convert sucrose into structural or defensive polysaccharides to enhance their stress resistance. In contrast, CA showed significantly higher levels of starch and sucrose, and lower levels of hemicellulose and water-soluble polysaccharides, suggesting a preference for converting sucrose into starch for nutrient storage rather than synthesizing structural polysaccharides. Additionally, AR contained some free monosaccharides, primarily sorbose and glucose. The content of free sorbose was significantly higher in CA than in WA and IWA (**Figure 3G**), while there was no significant difference in glucose content (**Figure 3H**). Liquid chromatography profiles and methodological investigations of sucrose, sorbose and glucose were presented in **Supplementary Figure 3** and **Supplementary Table 3**.

Finally, saccharides could significantly influence the contents of water-soluble and ethanol-soluble extractions. The water-extraction contents in WA and IWA were 10% lower than that in CA (**Figure 3I**), primarily caused by sucrose. Additionally, the ethanol-extraction in WA was lower than in CA, but remarkably higher than in IWA (**Figure 3J**). This phenomenon may be attributed to the higher levels of certain less polar components in WA (such as oligosaccharides) than IWA. Furthermore, as per the *Pharmacopoeia of the People’s Republic of China*, the water-extraction content in AR should exceed 17.0%. WA, IWA, and CA all meet this requirement.

#### 3.1.5 Differences in the contents of ten active compounds

**Figure 4:**
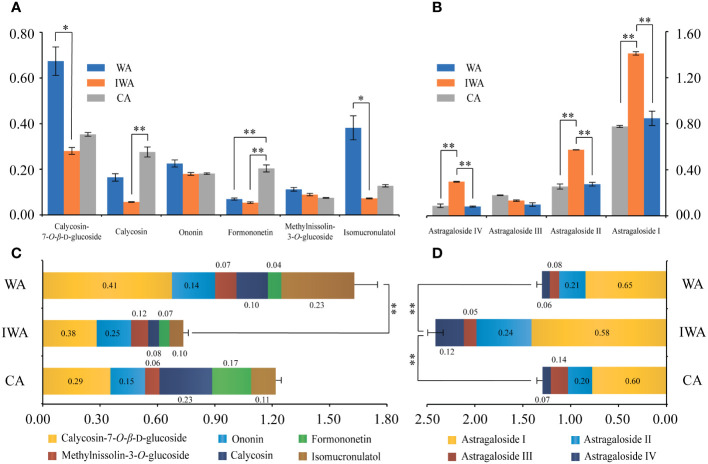
The contents of ten active compounds in WA, IWA and CA (mg/g). **(A)** Contents of six isoflavonoes, **(B)** Contents of four saponins, **(C)** Cumulative content of six isoflavonoes, **(D)** Cumulative content of four saponins. WA, Wild Astragali Radix; IWA, Imitated-wild Astragali Radix; CA, Cultivated Astragali Radix. **, *p*<0.01; *, *p*<0.05.

Flavonoids and saponins constituted the primary pharmacologically active compounds in AR (Wang et al., 2023). Analysis of total-flavonoids content revealed significantly higher levels in WA compared to IWA and CA (**Figure 3K**). Among these, ononin, formononetin, calycosin-7-*O*-*β*-D-glucoside, calycosin, methylnissolin-3-*O*-glucoside and isomucronulatol were widely reported pharmacologically active substances. Their quantitative results (**Figure 4A**) indicated higher levels of calycosin-7-*β*-D-glucoside and isomucronulatol in WA, higher levels of formononetin and calycosin in CA, and similar ononin and methylnissolin-3-*O*-glucoside levels across the three types of AR. The cumulative content of the six isoflavonoes followed the order: WA > CA > IWA (**Figure 4C**). These isoflavonoes could be classified into glycosides (calycosin-7-*O*-*β*-D-glucoside, ononin, methylnissolin-3-*O*-glucoside) and aglycones (calycosin, formononetin, isomucronulatol). The ratio of glycosides/aglycones could serve as a good indicator of AR growth and developmental status as well as its stress resistance (Chen et al., 2022). In WA, IWA and CA, the ratio of glycosides/aglycones stood at 1.63, 1.86 and 0.98, respectively. This indicated a higher trend of glycoside synthesis over aglycones in WA and IWA, while CA displays the opposite trend.

As for saponins, there was no significant difference in total saponin content among WA, IWA and CA (**Figure 3L**). Astragalosides I-IV were the main saponin in Astragalus (Dai et al., 2022; Yang et al., 2023a). Their quantitative results revealed that except for astragaloside III, the contents of astragaloside I, II, and IV were significantly higher in IWA compared to WA and CA (**Figure 4B**). The cumulative content of astragalosides I-IV was also highest in IWA (**Figure 4D**). Astragalosides I-IV exhibited pharmacological effects such as anti-aging and immune regulation (Wang et al., 2023), suggesting that IWA may have better efficacy in these aspects. Liquid chromatography profiles and methodological investigations of the ten compounds were presented in **Supplementary Figure 4** and **Supplementary Table 4**.

#### 3.1.6 Multi-data correlation analysis of WA, IWA and CA

**Figure 5:**
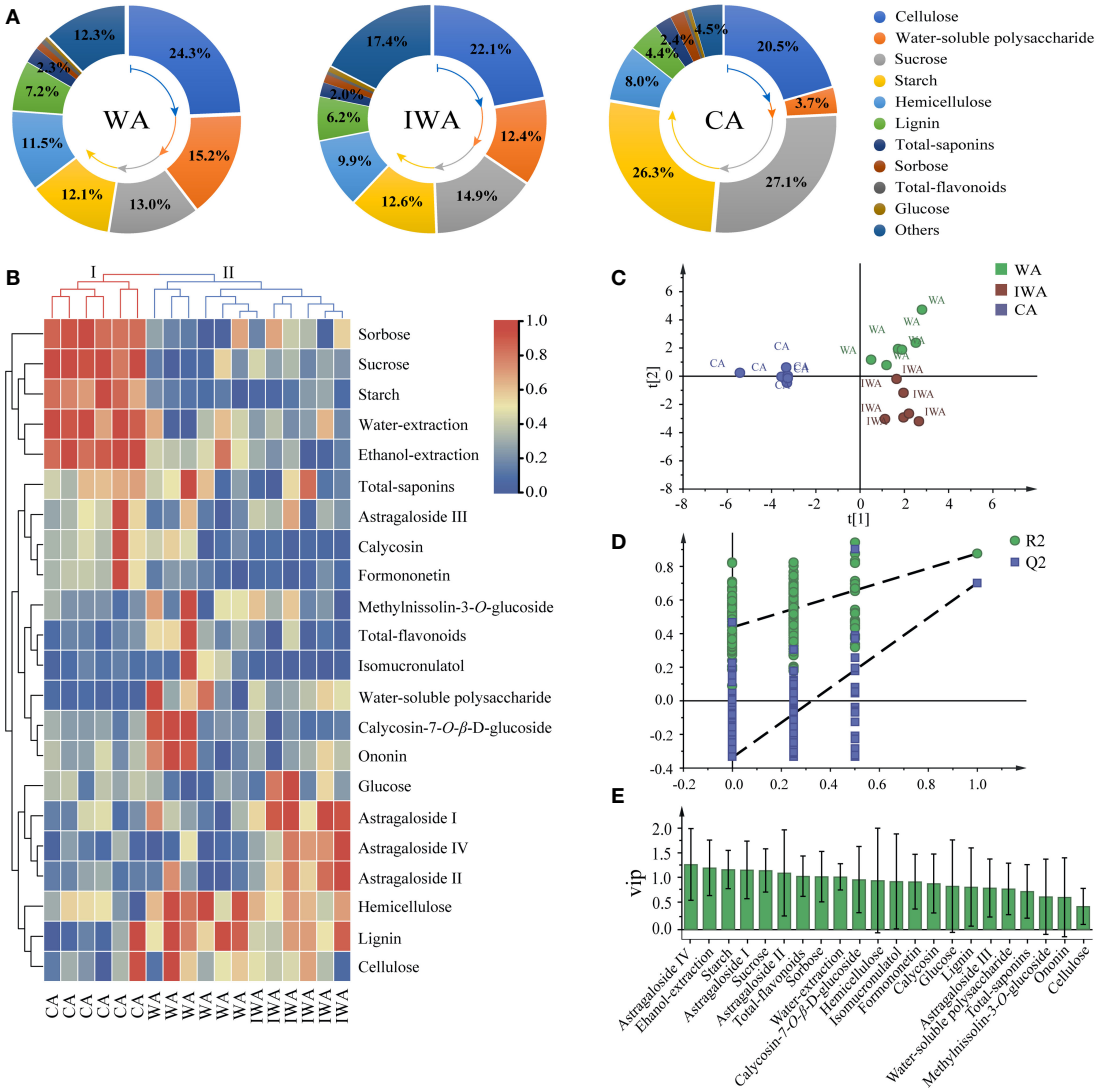
Correlation analysis of WA, IWA and CA. **(A)** Comparative charts of the chemical compositions, **(B)** Clustering heatmap, **(C)** The score plot of PLS-DA model, **(D)** Permutation test, **(E)** the plot of VIP values.

Firstly, we constructed comparative charts of the chemical compositions of WA, IWA and CA (**Figure 5A**). It was evident that three types of AR were predominantly composed of sugar components, but varied in the proportions. The main components of WA were cellulose, water-soluble polysaccharides, sucrose, starch and hemicellulose, with fewer lignin, total-flavonoids and total-saponins. The composition of IWA was essentially consistent with that of WA, with water-soluble polysaccharides constituting over 10% and the proportions of sucrose and starch being less than 30%. However, in CA, there was a notable decrease in water-soluble polysaccharides and a significant increase in the proportions of sucrose and starch. The cultivation and transplanting process of CA involved interventions such as watering and fertilization, leading to vigorous growth of CA. The enrichment of sucrose and starch, coupled with the decrease of water-soluble polysaccharides, resulted in substantial chemical composition changes between CA and WA. Additionally, there was still 10-20% of other components present in both WA and IWA. It was speculated that these components mainly include water, proteins, pectin, amino acids, and inorganic compounds. The content of these components was lower in CA, accounting for less than 5%. In summary, in terms of chemical composition, IWA is relatively consistent with WA, while CA exhibits significant differences from both.

Secondly, we drew a clustering heatmap (**Figure 5B**) by intergroup linkage, using euclidean square distance as the interval. CA was grouped into cluster I individually, showing a significant enrichment trend in sorbitol, sucrose, starch, extractions, and total-saponins. WA and IWA were grouped into cluster II, both exhibited significant enrichment in lignin, cellulose, and hemicellulose, with lower content of sugar components like sucrose, starch, sorbose. This indicated a consistency in chemical component content between WA and IWA, suggesting their close similarity in quality, distinct from CA. Furthermore, in terms of intragroup similarity, IWA and CA showed higher intragroup similarity, whereas WA exhibited greater variation within the group. For instance, there were significant differences in the content of secondary metabolites such as calycosin-7*-O-β*-D-glucoside, ononin and methylnissolin-3-*O*-glucoside between different samples of WA. This variation may be attributed to the greater genetic diversity, environmental conditions, and stress response mechanisms inherent in WA.

Finally, we established a PLS-DA model to further characterize the compositional differences among WA, IWA and CA. The parameters of PLS-DA model were R^2^Y=0.902 and Q^2 = ^0.794, indicating a well-fitted model with reliable clustering results (**Figure 5D**). The intra-group aggregation and inter-group separation effects were pronounced, with WA and IWA clustered on the right side of the Y-axis, while CA was uniquely distributed on the left side of the Y-axis (**Figure 5C**). The differential components screened by VIP > 1 mainly included starch, sucrose, sorbose, total-flavonoids, astragaloside I, astragaloside II, astragaloside IV (**Figure 5E**). Combined with the heatmap, it was evident that sugar components were primarily enriched in CA, with total-flavonoids abundant in WA and astragalosides enriched in IWA. Sugars such as sucrose, starch, and sorbose, as differentiating compounds in CA, could be served as markers for distinguishing CA from WA and IWA.

In summary, the chemical composition of IWA and WA was comparatively similar, including the levels and ratios of cellulose, hemicellulose, lignin, sucrose, starch, water-soluble polysaccharides, total-saponins and so on. However, the content of sucrose, starch and sorbose in CA was significantly higher than WA, indicating significant quality differences between them. Therefore, we concluded that IWA and WA were similar in quality, and recommended IWA to be the substitute of WA.

### 3.2 Multidimensional comparison of IWA at different growth years

#### 3.2.1 Differences in traits and microstructure

**Figure 6:**
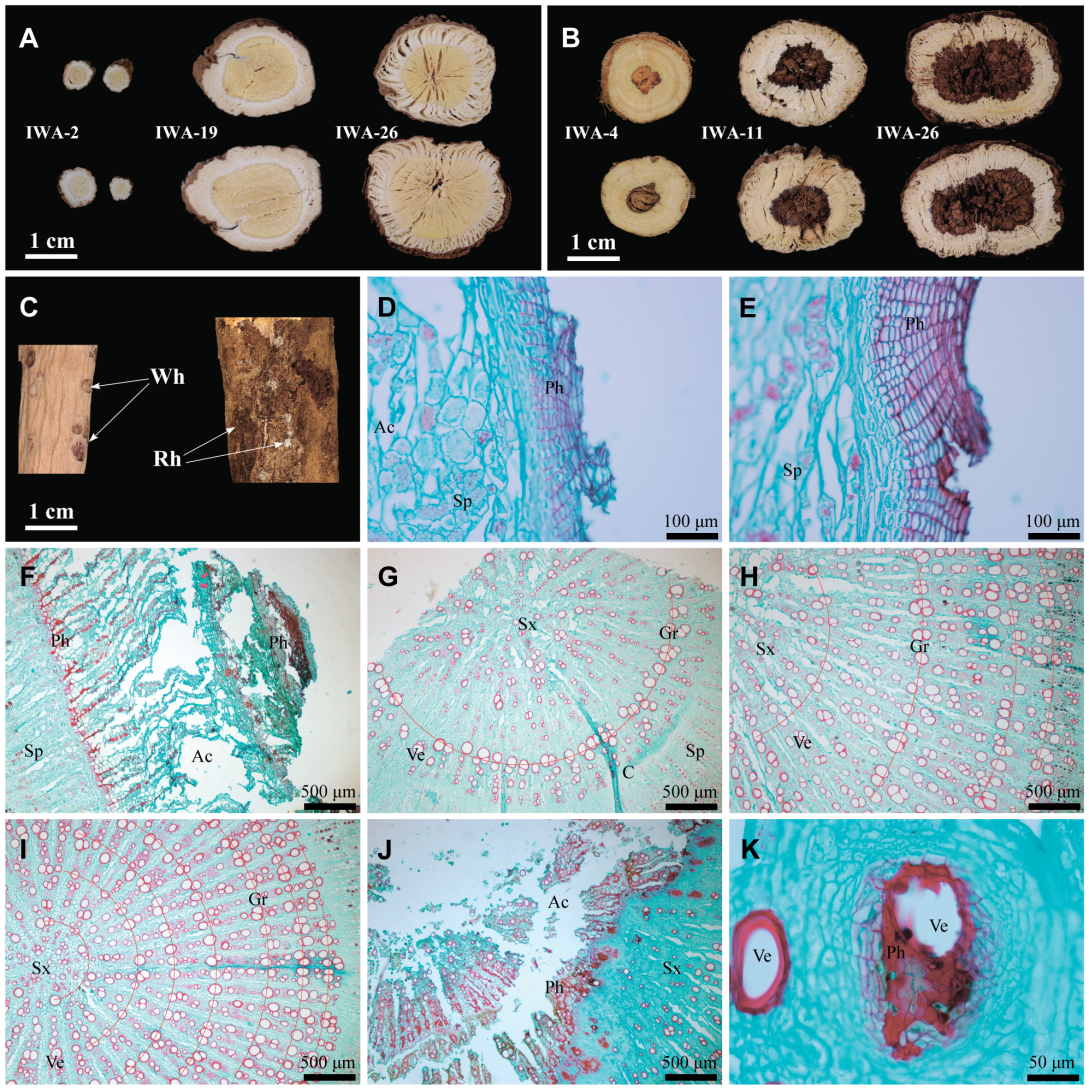
Macroscopic and microstructural characteristics of IWA at different growth years. **(A)** Cross-sections, **(B)** Cross-sections of heartwood, **(C)** Rhytidome and worm-hole, **(D)** Phellem of IWA-2, **(E)** Phellem of IWA-6, **(F)** Phellem of IWA-32 **(G)** Secondary-xylem of IWA-1, **(H)** Secondary-xylem of IWA-6, **(I)** Secondary-xylem of IWA-11, **(J)** Microstructure of heartwood, **(K)** Vessels in the heartwood. Rh, Rhytidome; Wh, Worm-hole; Ph, Phellem; Ac, Aerenchyma; Sp, Secondary-phloem; Sx, Secondary-xylem; C, Cambium; Gr, Growth rings; Ve, Vessel.

With the increasing growth duration, characteristics of IWA changed in external morphology, cross-section and microstructure. Firstly, the root diameter of IWA increased with ages, reaching a maximum diameter of 30 mm in IWA-25 (**Supplementary Figure 5A**). The root length showed rapid growth from 1 to 5 years, ranging from 50 cm to 120 cm, and stabilized in the range of 120-150 cm for the subsequent 6-32 years (**Supplementary Figure 5B**). The external characteristics of IWA were similar across different growth durations, exhibiting longitudinal wrinkles, lenticels, rhytidome and worm-holes. However, 1-5 years old IWA had fewer rhytidome, with the skin appearing soil-yellow. In contrast, the number and degree of rhytidome increased annually from 6 to 32 years, with the most pronounced bark decay observed in 25-32 year and presented a black-brown color (**Figure 6C**; **Supplementary Figure 6**). Considering the diameter, length and external characteristics, the shorter growth duration of 1-5 years resulted in slightly smaller root size, while the prolonged growth duration of 25-32 years led to more severe decay. IWA aged 6-20 years demonstrated stable characteristics.

Secondly, it could be observed that IWA of different growth years showed the characteristic ‘JinjingYulan’ (**Figure 6A**). IWA began to showed apparent heartwood from the fourth year onwards, and the size and degree of decay in the heart rot region increased with the growth duration (**Figure 6B**). The contents of flavonoids and saponins in the heartwood region of AR was lower than in the non-decayed region (Yin et al., 2019). The medicinal substance content of high-year-old IWA with severe decay may be affected.

Finally, from a microscopic perspective, there were noticeable changes in the phellem structure of IWA with increasing growth duration, characterized by significant intensification of phellem lignification and fragmentation of phellem (**Supplementary Figure 7**). IWA aged 1-5 years typically exhibited approximately 6-15 layers of phellem cells (**Figure 6D**), but the number increased to around 10-30 layers in the 6-20 years old IWA with a gradual intensification of lignification, leading to a deepening purple-red staining of phellem cells (**Figure 6E**). IWA aged 25-32 years displayed approximately 30-50 layers of phellem cells, with severe lignification in the outermost cells leading to a fragmented appearance in the outer phloem (**Figure 6F**). Growth rings were a feature identified for age determination of IWA (Peng et al., 2017), resulting from differential growth rates of xylem vessels in summer and winter. With increasing growth duration, the numbers of growth rings in IWA steadily increased (**Figures 6G–I**). Furthermore, in 4 years old IWA and above, the heartwood region exhibited apoptosis of secondary xylem cells caused by lignified tissue (**Figure 6J**). In some instances, remnants of lignified fillings in vessels and infiltration of surrounding cells by lignified tissue could be observed in the secondary xylem of high-year-old IWA (**Figure 6K**). The microscopic structural information of IWA at different growth durations were presented in **Supplementary Figure 8**.

In summary, IWA exhibited phase-specific changes in external characteristics and microscopic features with increasing growth duration. IWA aged 1-5 years was a period of rapid growth, with increasing diameter and length, fewer layers of rhytidome, and minimal or absent heartwood. IWA aged 6-20 years represented a stable growth phase, with a slowed increase in length, stable diameter within a certain range, an increase in the number of rhytidome, enhanced lignification compared to 1-5 year, and a slight rise in the degree of heartwood. IWA aged 25-32 years signified an aging growth phase, with slightly higher diameter and root length compared to 6-20 year but evident decay in the rhytidome and heartwood regions. Single plant root volume, biomass, and AR yield per acre were higher when the diameter and length were greater and the degree of decay was smaller (Huang et al., 2017). IWA aged 6-20 years outperformed 1-5 years old IWA with greater diameter and root length, and exhibited lower decay compared to 25-32 years old IWA. Therefore, IWA aged 6-20 years demonstrated advantages in terms of individual plant biomass and yield per acre.

#### 3.2.2 Differences in the contents of cell wall components and saccharides

**Figure 7:**
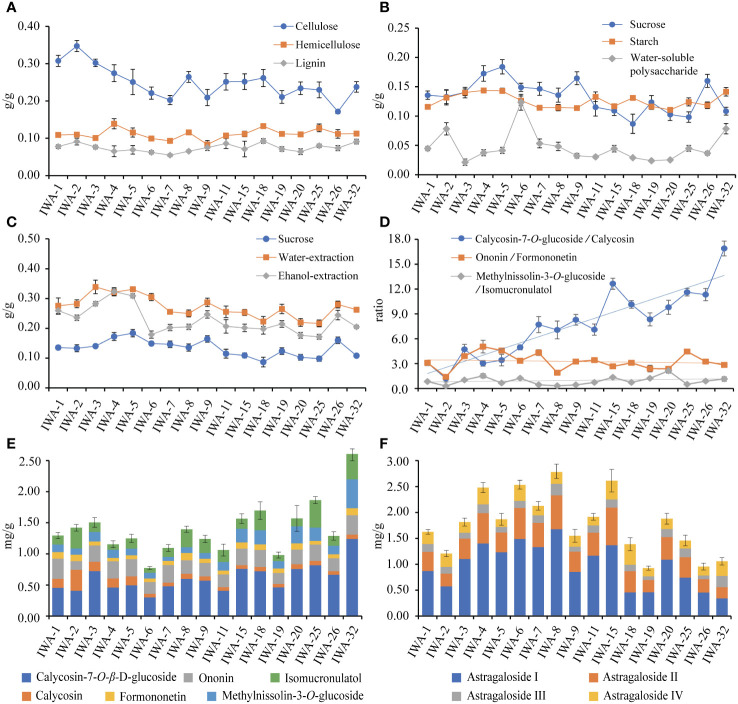
The contents of cell wall components and saccharides at different growth years of IWA. **(A)** Contents of cellulose, hemicellulose and lignin, **(B)** Contents of sucrose, starch and water-soluble polysaccharide, **(C)** Contents of sucrose, water-extraction and ethanol-extraction, **(D)** Ratios of isoflavonoes’ glycosides and aglycones, **(E)** Cumulative content of six isoflavonoes, **(F)** Cumulative content of four saponins.

The cellulose contents of 1-32 years old IWA ranged from 20% to 35%. The cellulose content was slightly higher in 1-5 years, reached a minimum around 6-7 years, gradually increased and stabilized at 6-32 years (**Figure 7A**). This phenomenon was related with the growth process of IWA. At 1-3 years, the diameter and length of IWA increased rapidly, and cell division was vigorous. Cellulose was the main component of the secondary cell wall and thus had a higher content during this period. A relative reduction in cellulose content was observed at 3-5 years due to the elevated accumulation of saccharide. Beyond 6 years, the root structure stabilized and the cellulose content tended to stabilize within a certain range. Hemicellulose and lignin exhibited lower content compared to cellulose, approximately 5-15%, and the content of both remained relatively stable across different growth years of IWA (**Figure 7A**).

The variations in sucrose, starch, and water-soluble polysaccharides among WA, IWA, and CA were noteworthy. It was of great significance to characterize the change rule of their contents with growth years. The patterns of sucrose and starch were generally consistent, showing an upward trend in the 1-5 years, a decline in the 6-20 years, and a subsequent upward trend from 25-32 years (**Figure 7B**). The water-soluble polysaccharides followed an increase in 1-2 years, a decrease from 3-5 years, reached the highest point at 6 years, and then stabilized around 5% from 6-32 years (**Figure 7B**). The variations in sucrose and water-soluble polysaccharide content would influence the content of extractions, with water-soluble and alcohol-soluble extractions exhibited patterns similar to sucrose (**Figure 7C**). An anomalous decrease in ethanol-extraction was observed at 2 and 6 years, likely attributed to the increased water-soluble polysaccharides that were insoluble in ethanol-extraction (**Figure 7B**). Additionally, the content of free glucose was lower at 1-5 years, increased at 5-7 years, and subsequently stabilized. The content of free sorbose showed no significant changes with increasing growth duration (**Supplementary Figure 9A**).

In conclusion, the content variations in cell wall components and saccharides were closely related to the developmental stages of IWA. During the rapid growth phase of IWA (1-5 years), the content of cellulose, sucrose, starch, water-extraction, and ethanol-extraction were relatively high. Moreover, the elevated levels of sucrose and starch indicated strong photosynthetic capabilities and vigorous growth characteristics during this period. In the stable growth phase of IWA (6-20 years), cellulose, hemicellulose, lignin, starch, and water-soluble polysaccharides remained stable within a certain range. This suggested that the growth status of IWA was relatively consistent during this period, with minimal fluctuations in growth and development. In contrast to the stable growth phase, the aging growth phase of IWA (25-32 years) showed an increase in the content of sucrose, starch, and water-soluble polysaccharides, resembling the levels observed in 1-5 years. This increase might be attributed to the heightened decay in the 25-32 years IWA, resulting in a further slowdown in growth and a reduction in the metabolic accumulation of sugar compounds.

#### 3.2.3 Differences in the contents of ten active compounds

The accumulation of six isoflavonoes showed a fluctuating upward trend from 6 to 32 years, reaching its peak at 32 years (**Figure 7E**). Calycosin-7-*O*-*β*-D-glucoside constituted the highest proportion, and its trend aligned consistently with the overall isoflavonoes accumulation. The levels of ononin and formononetin remained relatively stable across different durations, while calycosin-7-*O*-*β*-D-glucoside and calycosin contents were higher in 18-32 years old IWA. As reported, the ratio of glycosides/aglycones might be associated with their growth duration (Hao et al., 2023). In this study, the ratio of calycosin-7-*O*-*β*-D-glucoside/calycosin showed a significant positive correlation with the growth duration of IWA (**Figure 7D**), suggesting that it could serve as a potential auxiliary tool for determining IWA ages. However, the ratio of ononin/formononetin did not show apparent correlations.

The accumulation of astragaloside I-IV showed a fluctuating upward trend at 1-8 years and a subsequent decrease at 9-32 years (**Figure 7F**). The trends of astragaloside I, astragaloside II and astragaloside IV were relatively consistent with the overall cumulative trend, with higher levels observed at 4, 6, and 8 years. On the other hand, the content of astragaloside III remained relatively stable across different durations (**Figure 7F**). The increased content of saponins observed in 4-8 years old IWA may be attributed to the cross-sectional ratio of phloem. As growth progresses, there is an augmentation in heartwood development accompanied by a decline in phloem proportion. Given that saponins predominantly localize within the phloem, the content of saponins distributed in the phloem diminishes with age.

#### 3.2.4 Multi-data correlation analysis of IWA at different growth years

**Figure 8:**
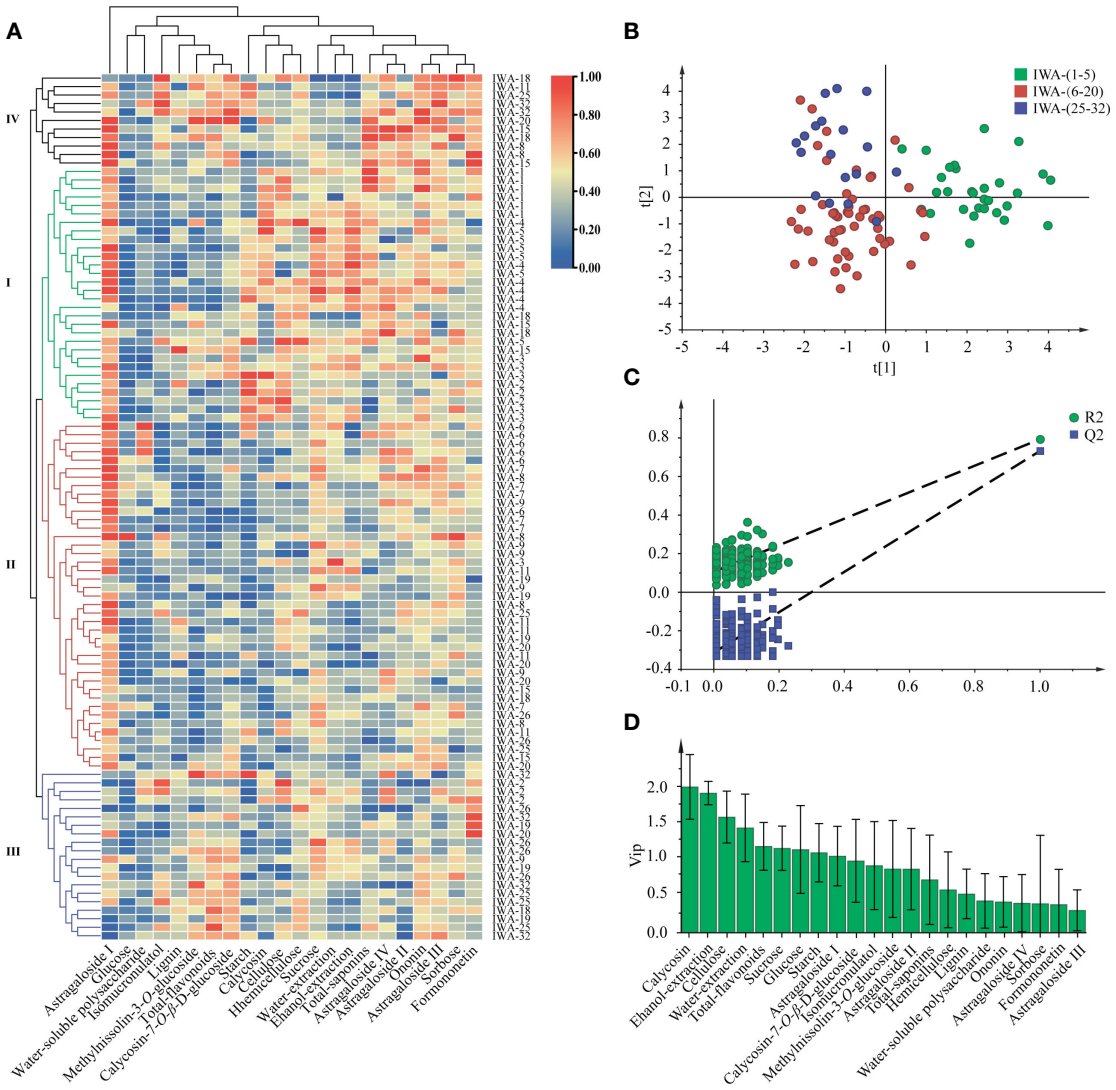
Correlation analysis of IWA at different growth years. **(A)** Clustering heatmap, **(B)** The score plot of PLS-DA model, **(C)** Permutation test, **(D)** The plot of VIP values.

Firstly, the clustering heatmap showed that IWA samples could be classified into four groups: Group I (1-5 years), Group II (6-20 years) and Group III (25- 32 years), while Group IV represented a few outlier samples (**Figure 8A**). It was reasonable to consider 6 and 20 years as critical time points of IWA quality changes. In terms of component clustering, similar components exhibited coordinated trends, such as starch, water-soluble polysaccharides, cellulose, and hemicellulose.

Subsequently, based on the aforementioned rule, PLS-DA analysis was performed based on three groups of IWA (1-5 years, 6-20 years, 25-32 years). The score plot indicated relatively concentrated samples within each group and a noticeable separation trend between groups (**Figure 8B**). The Q^2^ value of permutation test less than 0 confirmed the reliability of the model (**Figure 8C**). Calycosin-7-*O*-*β*-D-glucoside, ethanol-extraction, cellulose, and water-extraction were the main differentiating components screened by VIP > 1.2 (**Figure 8D**). Calycosin-7-*O*-*β*-D-glucoside was the component with the most significant difference, making it a potential marker for discerning 1-5 years, 6-20 years, and 25-32 years old IWA.

In summary, IWA aged 6-20 years reflected overall good quality with minimal variation over the growth years. They exhibited consistent characteristics, high yield, stable levels of cellulose, starch, water-soluble polysaccharides and sucrose, as well as relatively high contents of effective constituents. In contrast, IWA aged 1-5 years had slightly lower yield and higher sugar contents. IWA aged 25-32 years showed higher decay and lower saponins content, indicating weaker overall quality. Therefore, considering the quality and cost of IWA cultivation, we recommend to harvest IWA at 6-8 years.

## 4 Conclusion

The application of the multidimensional evaluation method established in this study yielded positive outcomes in evaluation of AR with different growth patterns and years. In the context of different growth patterns, the quality of IWA closely resembled WA in terms of apparent color, sectional structure, odor, thickness of phellem, diameter and number of vessels, morphology of phloem and xylem, cell wall components, water-soluble polysaccharide, flavonoid and saponin contents. But there were notable differences in quality between CA and WA. Therefore, we recommend using IWA as an alternative to WA rather than CA. For different growth years of IWA, the content of each component fluctuated more smoothly in 6-20 years old IWA, and the overall quality was superior. In contrast, 1-5 and 25-32 years old IWA exhibited some disadvantages, such as insufficient yield, significant withering, and a high content of sugary components. Considering both quality and economic aspects of planting, we recommended to harvest IWA after 6-8 years of cultivation. Furthermore, the multidimensional evaluation method we proposed could provide a scientific foundation for improving planting modes and determining planting years for other herbal species.

## Data availability statement

The raw data supporting the conclusions of this article will be made available by the authors, without undue reservation.

## Author contributions

YW: Writing – original draft, Writing – review & editing, Data curation, Investigation, Software, Conceptualization, Methodology, Resources, Validation, Visualization. CY: Data curation, Investigation, Writing – review & editing. JZ: Investigation, Resources, Writing – review & editing. YL: Investigation, Writing – review & editing. CT: Investigation, Writing – review & editing. JQ: Investigation, Writing – review & editing. TN: Methodology, Writing – review & editing. LK: Methodology, Writing – review & editing. YL: Conceptualization, Formal analysis, Supervision, Writing – review & editing. ZZ: Conceptualization, Funding acquisition, Project administration, Supervision, Writing – review & editing. LH: Conceptualization, Funding acquisition, Project administration, Supervision, Writing – review & editing.
