# Supplementary material for: An evaluation of Astragali Radix with different growth patterns and years, based on a new multidimensional comparison method
Source: Front Plant Sci. 2024 Feb 29;15:1368135. doi: 10.3389/fpls.2024.1368135 (PMC10937430; doi:10.3389/fpls.2024.1368135)
Supplement: Supplementary file 1 [file DataSheet_1.docx]

***Supplementary Material***

**Figures and Tables**

**Supplementary Figure 1.** Chemical structures of thirteen standard compounds.

**Supplementary Figure 2.** Diameter (A) and Length (B) of Wild Astragali Radix (WA), Imitated-wild Astragali Radix (IWA) and Cultivated Astragali Radix (CA).

**Supplementary Figure 3.** Ultra-high-performance liquid chromatography coupled with evaporative light scattering detector (UPLC-ELSD) chromatograms of sorbose (A), glucose (A), sucrose (B) of Astragali Radix.

**Supplementary Figure 4.** Ultra-high-performance liquid chromatography coupled with evaporative light scattering detector (UPLC-ELSD) chromatograms of ten active compounds of Astragali Radix.

**Supplementary Figure 5.** Diameter (A) and Length (B) of Imitated-wild Astragali Radix (IWA) at different growth years.

**Supplementary Figure 6.** Image of Imitated-wild Astragali Radix (IWA) at different growth years.

**Supplementary Figure 7.** Microscopic phellem cells of Imitated-wild Astragali Radix (IWA) at different growth years.

**Supplementary Figure 8.** Microscopic structure of Imitated-wild Astragali Radix (IWA) at different growth years.

**Supplementary Figure 9.** The Content of sorbose (A), glucose(A), total-flavonoids (B), total-saponins (B) in Imitated-wild Astragali Radix (IWA) at different growth years.

**Supplementary Table** **1.** The detailed information of Astragali Radix samples.

**Supplementary Table** **2.** Calibration curves, *R*^2^ and Linear Range of four components.

**Supplementary Table** **3.** Calibration curves, precision, repeatability, stability and recovery of three sugars.

**Supplementary Table** **4.** Calibration curves, precision, repeatability, stability and recovery of ten active compounds.


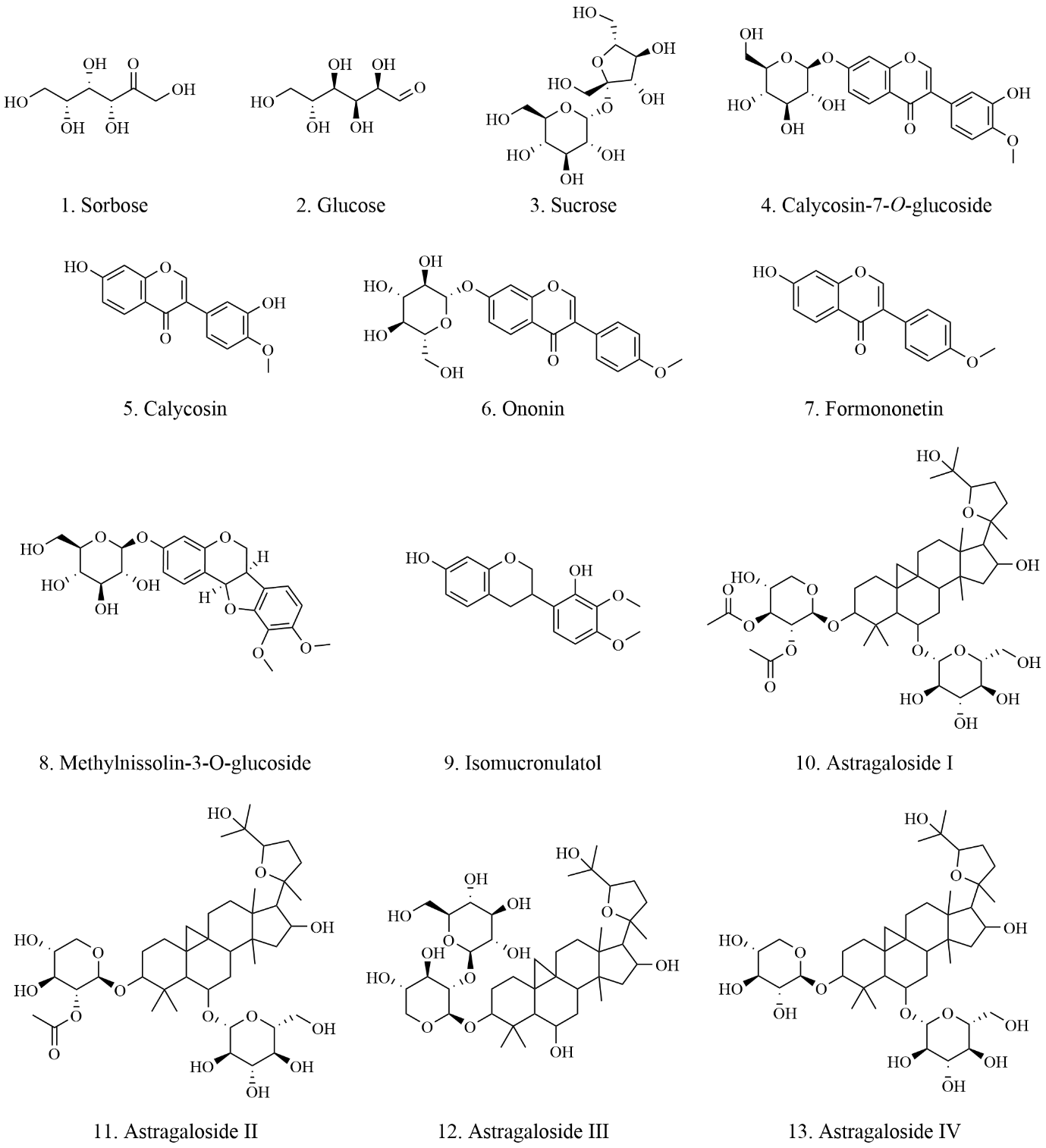


**Supplementary Figure 1.** Chemical structures of thirteen standard compounds.


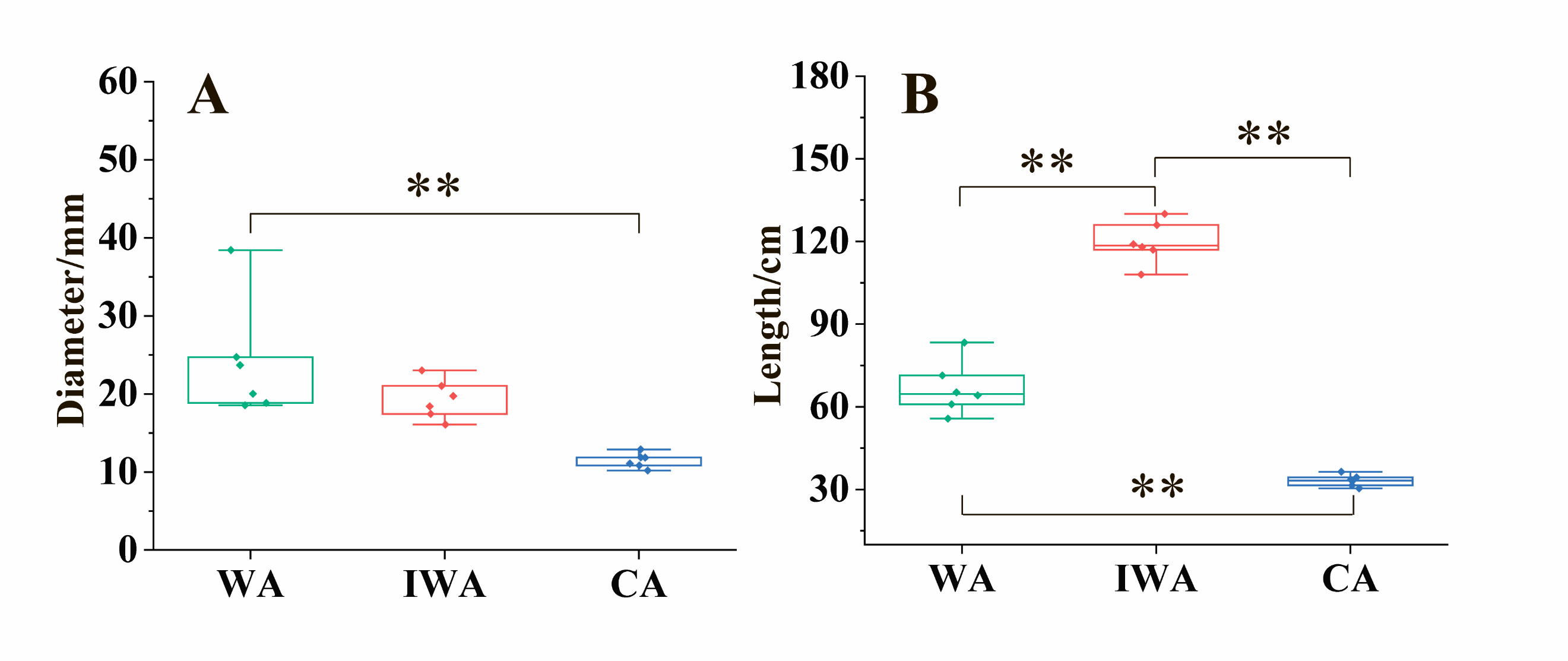


**Supplementary Figure 2.** Diameter (**A**) and Length (**B**) of Wild Astragali Radix (WA), Imitated-wild Astragali Radix (IWA) and Cultivated Astragali Radix (CA). **, *p* < 0.01.


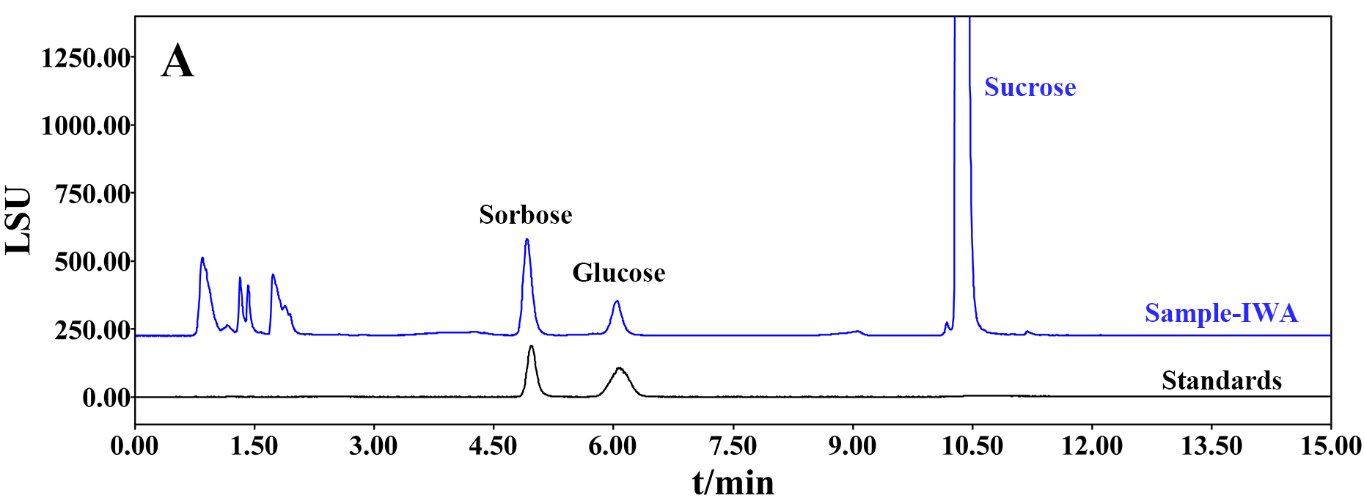

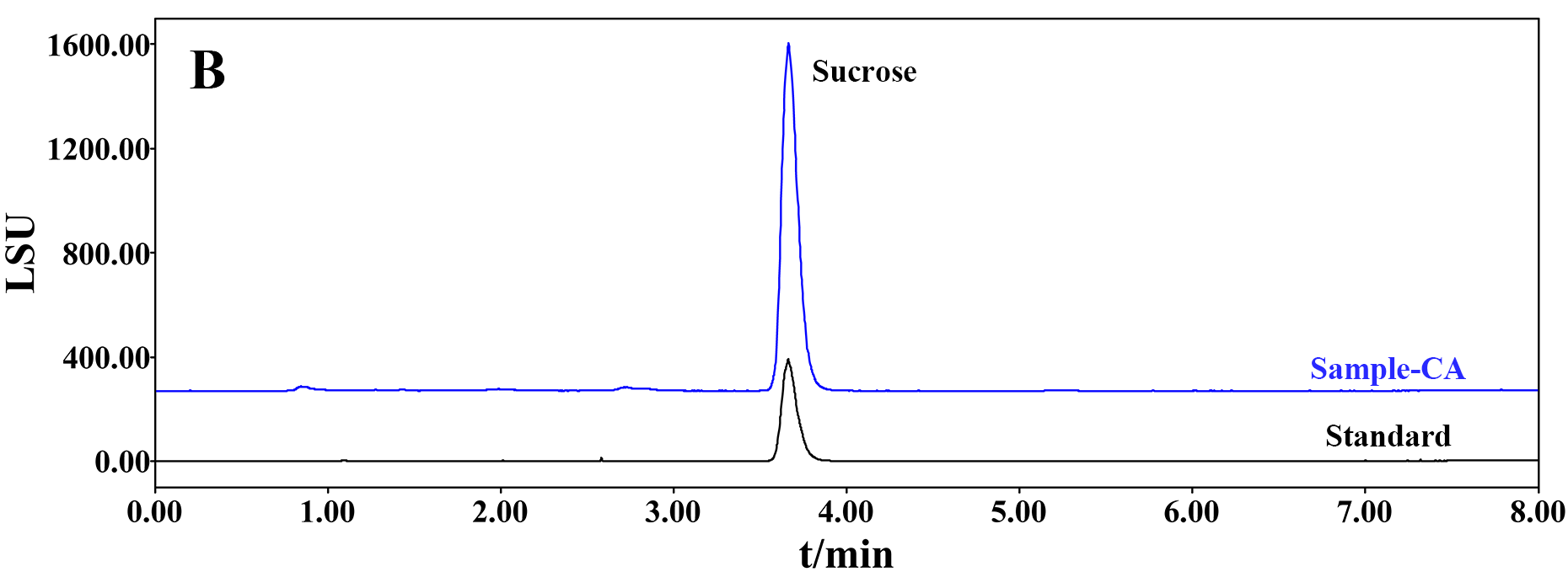


**Supplementary Figure 3.** Ultra-high-performance liquid chromatography coupled with evaporative light scattering detector (UPLC-ELSD) chromatograms of sorbose (**A**), glucose (**A**), sucrose (**B**) of Astragali Radix.


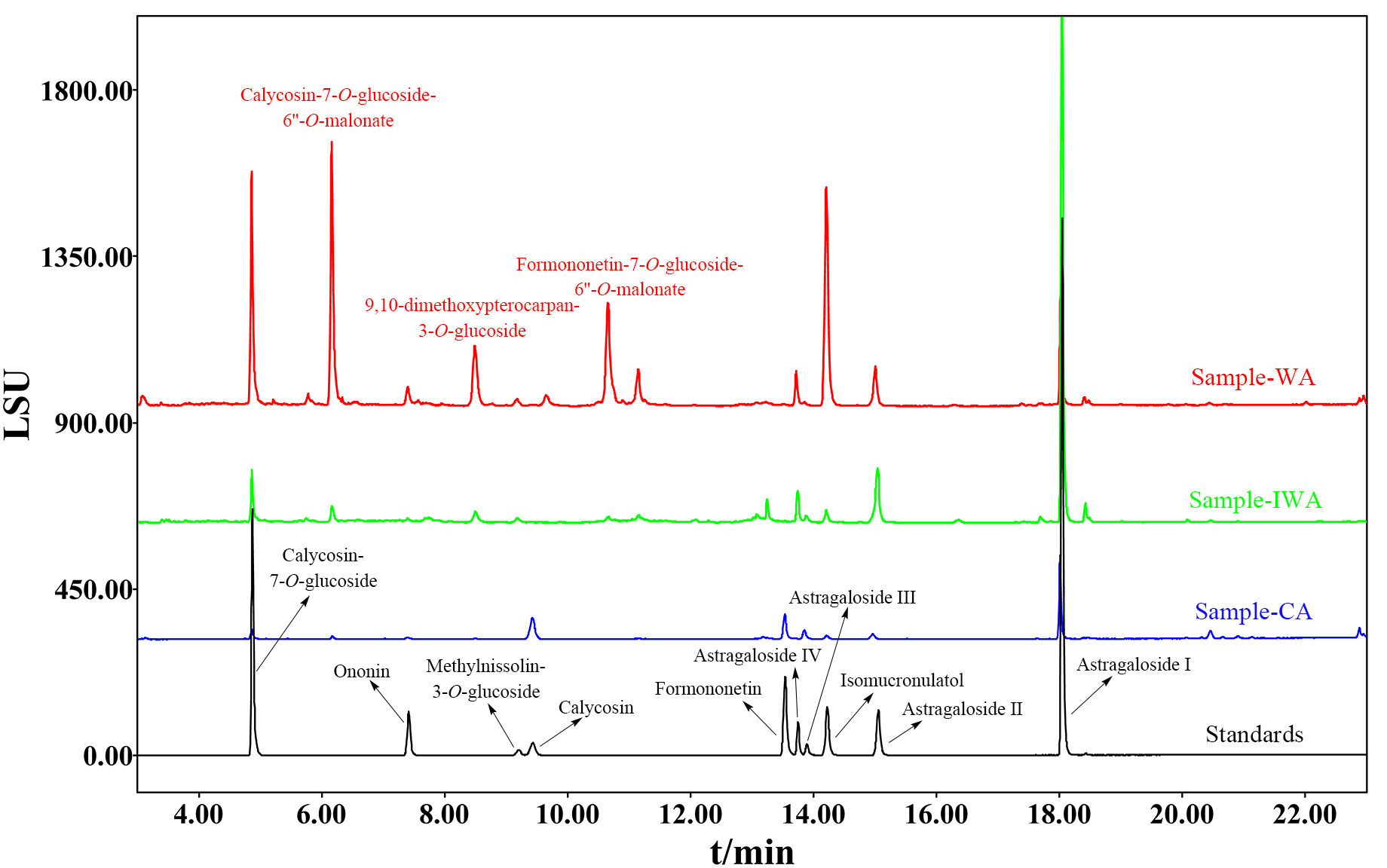


**Supplementary Figure 4.** Ultra-high-performance liquid chromatography coupled with evaporative light scattering detector (UPLC-ELSD) chromatograms of ten active compounds of Astragali Radix.

**
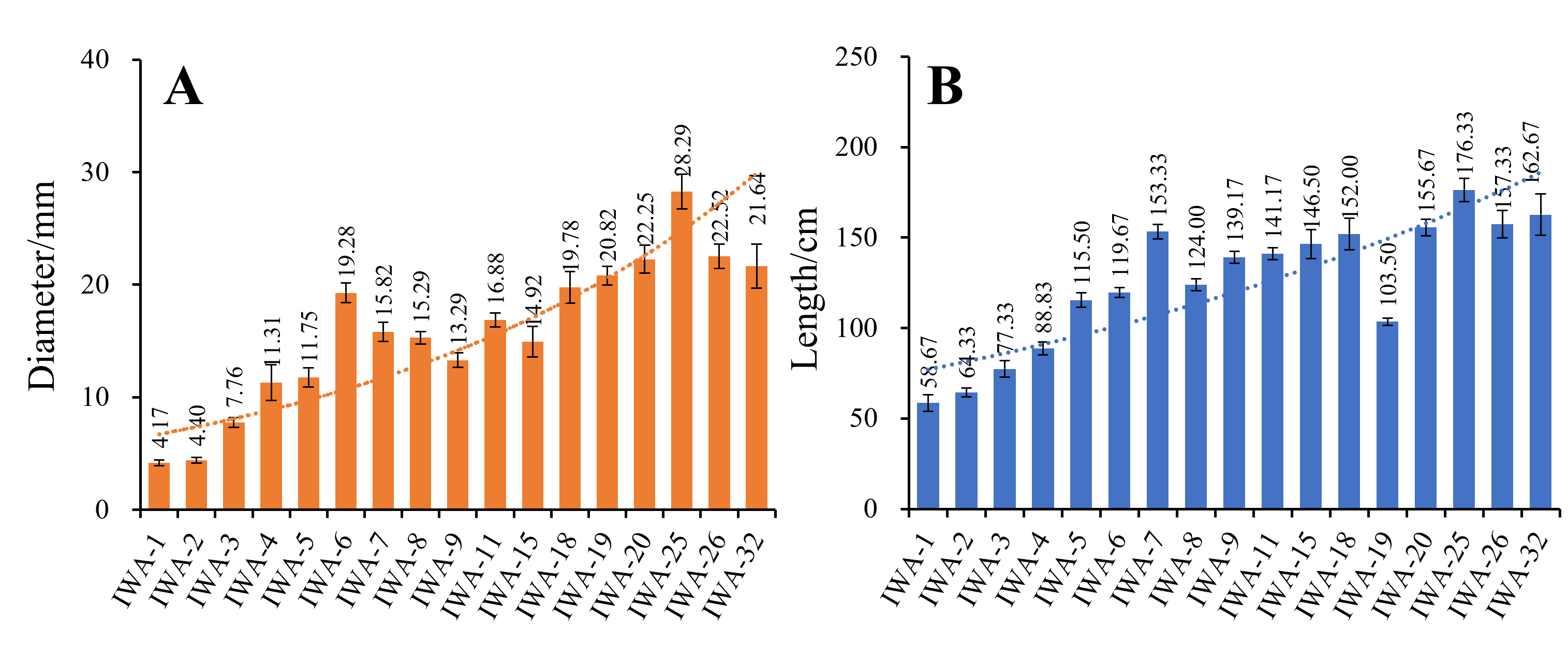
**

**Supplementary Figure 5.** Diameter (**A**) and Length (**B**) of Imitated-wild Astragali Radix (IWA) at different growth years.


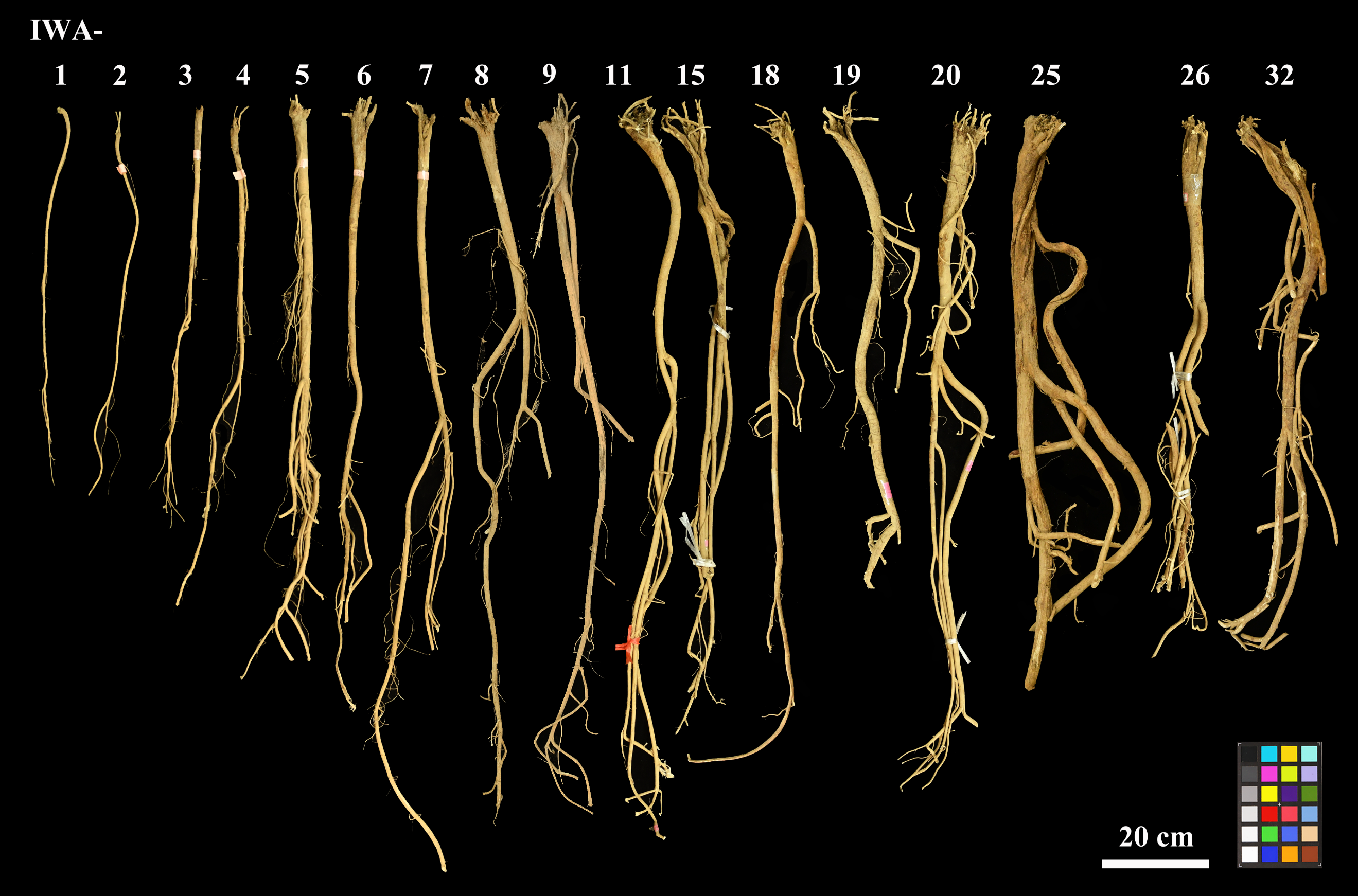


**Supplementary Figure 6.** Image of Imitated-wild Astragali Radix (IWA) at different growth years.


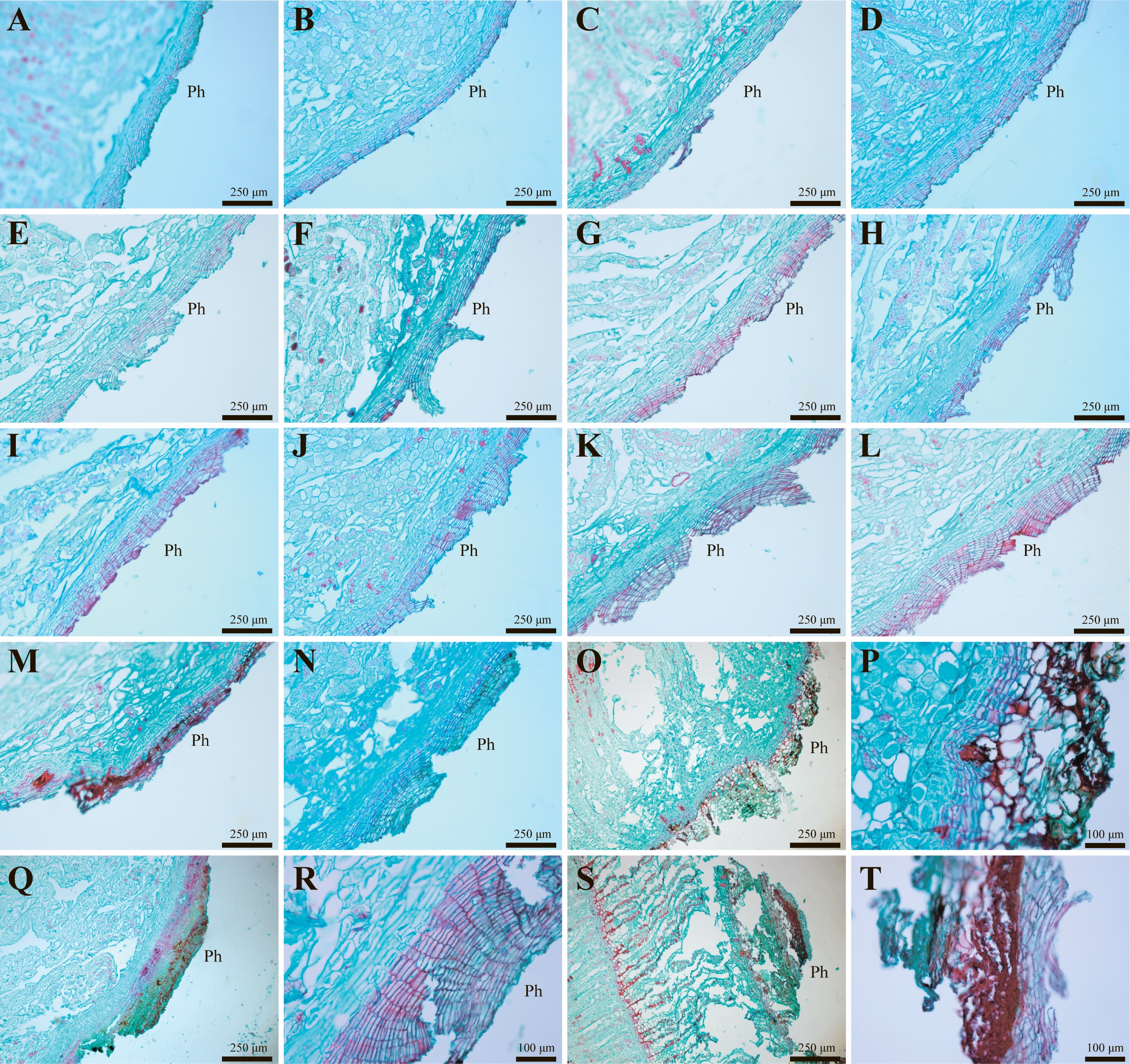


**Supplementary Figure 7.** Microscopic phellem cells of Imitated-wild Astragali Radix (IWA) at different growth years

(**A**) IWA-1, (**B**) IWA-2, (**C**) IWA-3, (**D**) IWA-4, (**E**) IWA-5, (**F**) IWA-6, (**G**) IWA-7, (**H**) IWA-8, (**I**) IWA-9, (**J**) IWA-11, (**K**) IWA-15, (**L**) IWA-18, (**M**) IWA-19, (**N**) IWA-20, (**O**) IWA-25, (**P**) Enlarged phellem cell of IWA-25, (**Q**) IWA-26, (**R**) Enlarged phellem cell of IWA-26, (**S**) IWA-32, (**T**) Enlarged phellem cell of IWA-32. Ph: Phellem.


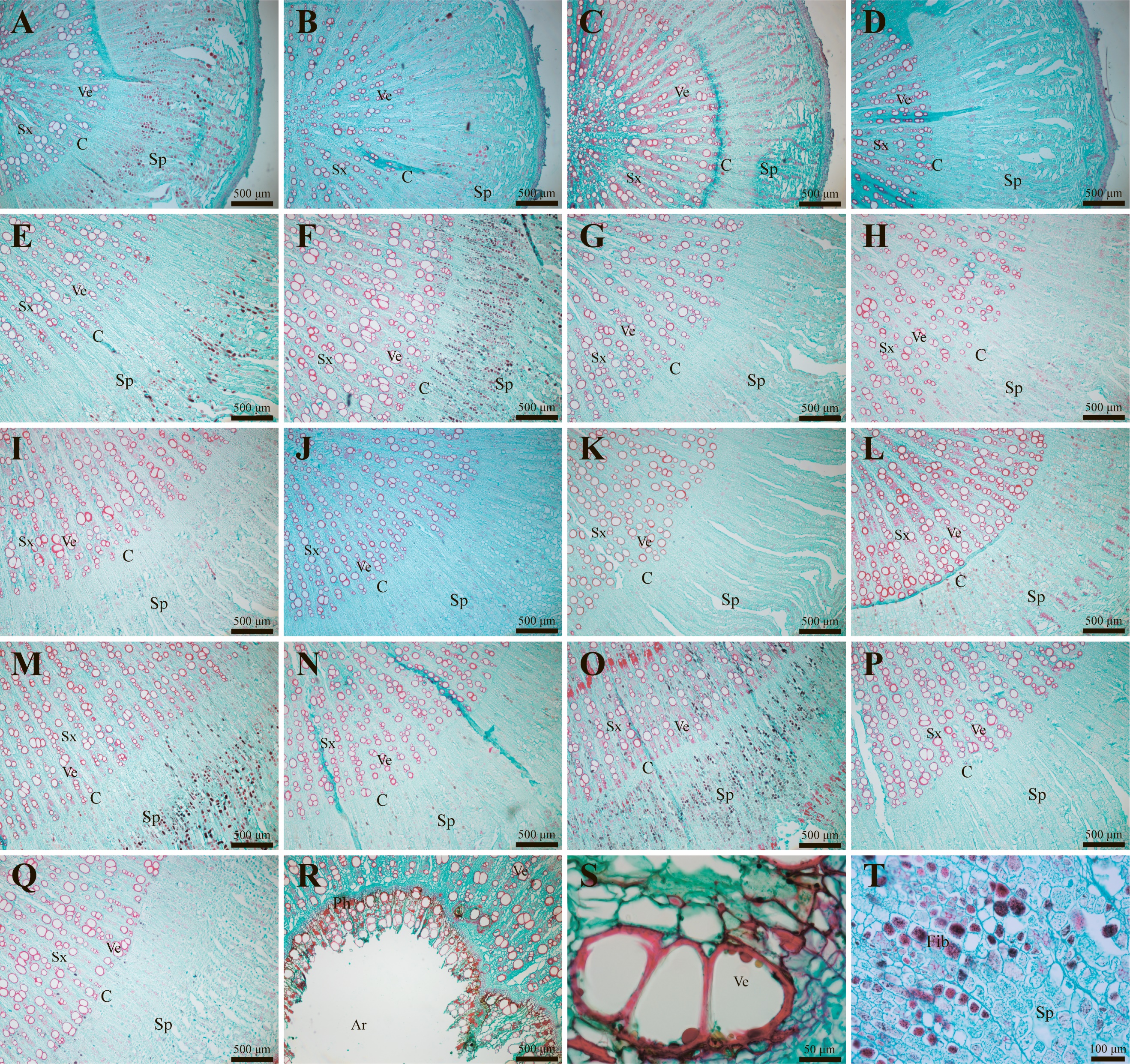


**Supplementary Figure 8.** Microscopic structure of Imitated-wild Astragali Radix (IWA) at different growth years

(**A**) IWA-1, (**B**) IWA-2, (**C**) IWA-3, (**D**) IWA-4, (**E**) IWA-5, (**F**) IWA-6, (**G**) IWA-7, (**H**) IWA-8, (**I**) IWA-9, (**J**) IWA-11, (**K**) IWA-15, (**L**) IWA-18, (**M**) IWA-19, (**N**) IWA-20, (**O**) IWA-25, (**P**) IWA-26, (**Q**) IWA-32, (**R**) Hollow heart and phellem cell ring of IWA-32, (**S**) Vessels in the phellem cell ring, (**T**) Lignified phloem parenchyma of IWA. Sx: Secondary-xylem; C: Cambium；Sp: Secondary-phloem; Ve: Vessel; Ph: Phellem; Ac: Aerenchyma; Fib: Fibrocyte.

**
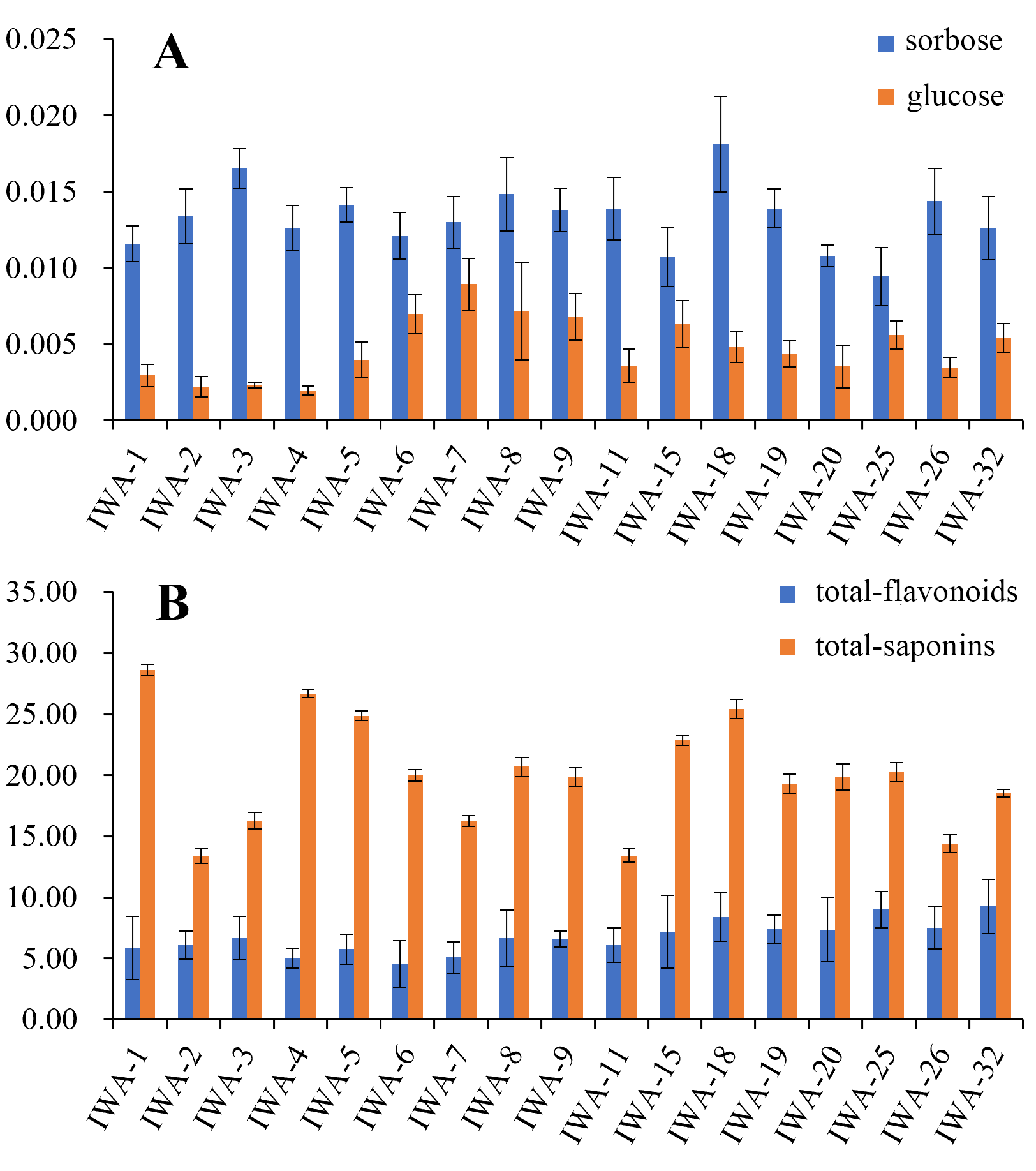
**

**Supplementary Figure 9.** The Contents of sorbose (**A**), glucose(**A**), total-flavonoids (**B**), total-saponins (**B**) in Imitated-wild Astragali Radix (IWA) at different growth years.

**Supplementary Table** **1.** The detailed information of Astragali Radix samples (*n*=6).

|  | Note | Growth years | Type | Collection Area | Collection Time |
| --- | --- | --- | --- | --- | --- |
| Different growth patterns of AR | WA | >10 | wild | Yingxian, Suozhou, Shanxi, China | 2020.10 |
|  | IWA | 6 | imitated-wild | Zizhou, Yulin, Shaanxi, China | 2020.10 |
|  | CA | 2 | cultivated | Minxian, Dingxi, Gansu, China | 2020.10 |
| Different growth years of IWA | IWA-1 | 1 | imitated-wild | Zizhou, Yulin, Shaanxi, China | 2020.10 |
|  | IWA-2 | 2 | imitated-wild | Zizhou, Yulin, Shaanxi, China | 2020.10 |
|  | IWA-3 | 3 | imitated-wild | Zizhou, Yulin, Shaanxi, China | 2020.10 |
|  | IWA-4 | 4 | imitated-wild | Zizhou, Yulin, Shaanxi, China | 2020.10 |
|  | IWA-5 | 5 | imitated-wild | Zizhou, Yulin, Shaanxi, China | 2020.10 |
|  | IWA-6 | 6 | imitated-wild | Zizhou, Yulin, Shaanxi, China | 2020.10 |
|  | IWA-7 | 7 | imitated-wild | Zizhou, Yulin, Shaanxi, China | 2020.10 |
|  | IWA-8 | 8 | imitated-wild | Zizhou, Yulin, Shaanxi, China | 2020.10 |
|  | IWA-9 | 9 | imitated-wild | Zizhou, Yulin, Shaanxi, China | 2020.10 |
|  | IWA-11 | 11 | imitated-wild | Zizhou, Yulin, Shaanxi, China | 2020.10 |
|  | IWA-15 | 15 | imitated-wild | Zizhou, Yulin, Shaanxi, China | 2020.10 |
|  | IWA-18 | 18 | imitated-wild | Zizhou, Yulin, Shaanxi, China | 2020.10 |
|  | IWA-19 | 19 | imitated-wild | Zizhou, Yulin, Shaanxi, China | 2020.10 |
|  | IWA-20 | 20 | imitated-wild | Zizhou, Yulin, Shaanxi, China | 2020.10 |
|  | IWA-25 | 25 | imitated-wild | Zizhou, Yulin, Shaanxi, China | 2020.10 |
|  | IWA-26 | 26 | imitated-wild | Zizhou, Yulin, Shaanxi, China | 2020.10 |
|  | IWA-32 | 32 | imitated-wild | Zizhou, Yulin, Shaanxi, China | 2020.10 |

WA: Wild Astragali Radix, IWA: Imitated-wild Astragali Radix, CA: Cultivated Astragali Radix.;

**Supplementary Table** **2.** Calibration curves, *R*^2^ and Linear Range of four components.

|  | compound | Calibration curves | *R*^2^ | Linear Range（mg/ml） |
| --- | --- | --- | --- | --- |
| Water-soluble polysaccharide | sucrose | y = 5.5848 x-0.0024 | 0.9999 | 0.05-0.50 |
| Starch | sucrose | y = 5.5362 x-0.0039 | 0.9997 | 0.01-0.10 |
| Total-Flavonoids | calycosin-7-*O*-*β*-D-glucoside | y = 11.514 x + 0.0048 | 0.9999 | 0.005-0.080 |
| Total-Saponins | astragaloside IV | y = 1.6035 x + 0.2178 | 0.9971 | 0.02-0.20 |

**Supplementary Table** **3.** Calibration curves, precision, repeatability, stability and recovery of three sugars.

| Sugar | Calibration curves | tR  (min) | *R*^2^ | Linear Range（mg/ml） | Precision (RSD, *n*=6) | Repeatability (RSD, *n*=6) | Stability  (RSD, *n*=6) | \| Recovery Mean \| \| --- \| | Recovery (RSD, *n*=6) |
| --- | --- | --- | --- | --- | --- | --- | --- | --- | --- | --- |
| sorbose | y = 1.9811 x + 6.5294 | 5.05 | 0.9999 | 0.103-2.070 | 1.96% | 2.28% | 1.44% | 100.69% | 2.96% |
| glucose | y = 1.8161 x + 6.9631 | 6.30 | 0.9999 | 0.058-1.165 | 1.54% | 2.87% | 2.53% | 98.18% | 3.58% |
| sucrose | y = 1.7244 x + 6.8567 | 3.66 | 0.9996 | 0.152-1.519 | 1.33% | 1.82% | 1.40% | 96.69% | 2.63% |

**Supplementary Table** **4.** Calibration curves, precision, repeatability, stability and recovery of ten active compounds.

| Compound | Calibration curves | tR  (min) | *R*^2^ | Linear Range（mg/ml） | Precision (RSD, *n*=6) | Repeatability (RSD, *n*=6) | Stability  (RSD, *n*=6) | \| Recovery Mean \| \| --- \| | Recovery (RSD, *n*=6) |
| --- | --- | --- | --- | --- | --- | --- | --- | --- | --- | --- |
| calycosin-7-*O*-*β*-D glucoside | y = 1.5870 x + 6.9048 | 4.87 | 0.9993 | 0.198-3.960 | 1.30% | 2.73% | 1.33% | 99.46% | 2.48% |
| ononin | y = 1.6130 x + 6.6520 | 7.41 | 0.9996 | 0.094-1.875 | 1.49% | 2.37% | 2.71% | 98.23% | 1.79% |
| methylnissolin-3-*O*-glucoside | y = 1.6190 x + 6.6590 | 9.22 | 0.9997 | 0.046-0.915 | 1.79% | 2.31% | 0.83% | 98.20% | 2.63% |
| calycosin | y = 1.6337 x + 6.9966 | 9.44 | 0.9995 | 0.054-1.070 | 2.47% | 2.92% | 2.56% | 97.23% | 3.31% |
| formononetin | y = 1.4800 x + 6.9026 | 13.54 | 0.9996 | 0.112-2.245 | 1.38% | 2.41% | 1.68% | 97.55% | 2.25% |
| astragaloside IV | y = 1.6046 x + 6.7887 | 13.73 | 0.9997 | 0.055-1.095 | 1.65% | 2.61% | 1.64% | 94.99% | 2.32% |
| astragaloside III | y = 1.2522 x + 6.2390 | 13.87 | 0.9989 | 0.046-0.920 | 4.13% | 3.70% | 3.22% | 102.80% | 4.54% |
| isomucronulatol | y = 1.6537 x + 7.0525 | 14.22 | 0.9997 | 0.085-1.690 | 1.64% | 2.40% | 1.52% | 99.77% | 2.19% |
| astragaloside II | y = 1.5784 x + 6.8846 | 15.03 | 0.9996 | 0.098-1.945 | 1.93% | 1.08% | 1.03% | 95.76% | 2.44% |
| astragaloside I | y = 1.6092 x + 6.5747 | 18.04 | 0.9999 | 0.252-4.035 | 0.85% | 0.80% | 0.54% | 97.45% | 1.22% |
